# Profiling of pollen in honey using multispectral imaging flow cytometry

**DOI:** 10.1038/s41538-026-01188-6

**Published:** 2026-10-30

**Authors:** Franziska Walther, Martin Hofmann, Martina Janke, Silvio Erler, Elsa Friedrich, Manuel Treder, Demetra Rakosy, W. Stanley Harpole, Patrick Mäder, Alicia Berdún, Thomas Hornick, Susanne Dunker

**Affiliations:** 1 Department Physiological Diversity, Helmholtz Centre for Environmental Research UFZ, Leipzig, Germany; 2 German Centre for integrative Biodiversity Research iDiv Halle-Jena-Leipzig, Leipzig, Germany; 3 Data-intensive Systems and Visualization Group (dAI.SY), Technische Universität Ilmenau, Ilmenau, Germany; 4 Lower Saxony State Office for Consumer Protection and Food Safety LAVES - Institute of Apiculture, Celle, Germany; 5 Institute for Bee Protection, Federal Research Centre for Cultivated Plants, Julius Kühn Institute (JKI), Braunschweig, Germany; 6 Zoological Institute, Technische Universität Braunschweig, Braunschweig, Germany; 7 University of Hohenheim, State Institute of Bee Research, Stuttgart, Germany; 8 Department Community Ecology, Helmholtz Centre for Environmental Research UFZ, Leipzig, Germany; 9 Thuenen Institute of Biodiversity, Braunschweig, Germany; 10 Martin Luther University Halle-Wittenberg, Halle, Germany; 11 Faculty of Biological Sciences, Friedrich Schiller University Jena, Jena, Germany; 12 CICYTTP (CONICET-Prov. ER-UADER), Laboratorio Interdisciplinario de Biología y Genética Molecular, Instituto de Botánica del Nordeste (CONICET-UNNE), Corrientes, Argentina

**Keywords:** Biological techniques, Computational biology and bioinformatics, Plant sciences

## Abstract

Pollen identification from honey (melissopalynology) is of great economic and consumer relevance. Traditional microscopy is time-consuming and dependent on expert knowledge, thus limiting high-throughput analyses. We tested whether pollen images generated with Multispectral Imaging Flow Cytometry (MIFC) classified by machine learning (ML) (convolution neural networks (CNNs) and random forest (RF)) can serve as a fast and reproducible method for identifying pollen in honey. We examined how the classification of honey pollen is affected by images from different matrices (anther and honey samples), how results compare with traditional microscopic analysis and used a set of human-interpretable image features to better explain ML results. The study focused on the six common target pollen in honey *Brassica*, *Castanea*, *Centaurea*, *Helianthus*, *Phacelia* and *Tilia* type. A classifier trained with a combination of anther and honey brightfield and fluorescence images provided the best predictions for 13 monofloral and 10 polyfloral honeys. Yielding pollen identification results comparable to microscopy, three times more pollen can be evaluated, and a measurement time of a maximum of 10 min, the method demonstrates its high potential as a powerful screening tool in melissopalynology.

## Introduction

The analysis of pollen grains in honey, also known as melissopalynology, is essential for determining the botanical and geographical origin of honey, as well as a component for detecting honey adulteration. The qualitative melissopalynology is based on the identification and counting of the plant particles (e.g. pollen grains) in the honey sediment, which serve as unique markers of the plants visited by honey bees^1–4^. This analysis is essential for distinguishing different honey types, including monofloral and polyfloral honeys. Polyfloral honeys originate from a mixture of nectar sources, while monofloral honeys are primarily produced from the nectar of one dominant plant species^5,6^ and number of pollen grains in selected monofloral honey allow an indirect estimation of the nectar proportion of the respective plant^7,8^. Monofloral honeys are highly valued for their unique characteristics, such as flavour^9,10^ or health benefits^11,12^. As a result, they often achieve a higher market price^8,13^. Identification of single pollen grains in monofloral and polyfloral honey serves also as a valuable matrix for environmental monitoring. As honey bees forage for nectar and pollen over large areas (approximately 10 km^2^), the resulting pollen composition in honey reflects the main floral composition and environmental conditions of the landscape^14,15^.

Manual microscopic pollen identification relies on the morphological characteristics of pollen, such as shape, size, surface texture, ornamentation and structure, to achieve accurate discrimination among taxa^2,16–21^. It remains the gold standard for melissopalynological analysis because it directly assesses these characteristics, although the method is highly time-consuming, expert-requiring and therefore costly. Identifying and counting pollen grains requires specialised, time-consuming training and a lot of practice^22–24^. The accuracy of microscopic pollen analysis depends to a large extent on the analyst’s expertise, the microscopical equipment and its optical quality, and working conditions like temporal constraints and psychological factors such as memory limitations or fatigue^25,26^. The achievable taxonomic resolution further depends on the morphological distinctiveness of the pollen types, as well as on the availability of reliable reference material and the laboratory’s software and data management systems^1,27^. Many taxa cannot be identified beyond the genus or family level because their pollen morphology shows only minor differences^26,28,29^. This issue is particularly pronounced in species belonging to the Brassicaceae^30–33^, Rosaceae^34–37^ or Asteraceae^38,39^ families. As a result, differences in the level of taxonomic resolution can affect data comparability and the interpretation of ecological or geographical patterns. These factors limit large-scale studies or high-throughput analyses. In melissopalynology analysis, an additional source of variation arises from the commonly over- and under-representation of certain pollen types, which strongly influences their relative abundance^1,18^.

Several non-optical methods have been developed for high-throughput pollen analysis, including metabarcoding and Fourier Transform Infrared (FTIR) spectroscopy to identify plant species. Despite offering high taxonomic resolution, DNA-based methods are usually not providing quantitative information about pollen abundance due to biases introduced during PCR amplification and sequencing^40–42^. FTIR spectroscopy analyses the molecular structure of pollen grains based on their characteristic infrared ‘fingerprint’^43,44^. However, the technique has limitations in taxonomic resolution, as the macromolecular composition within a family or genus does not differ sufficiently, meaning that similar species may produce overlapping spectra^45–47^.

In recent years, several advanced optical imaging techniques for analysing pollen in honey have been developed to enhance traditional manual microscopy by using pollen images to train specific AI classifiers (Table 1). Hence, they now enable automated, reproducible, fast and high-throughput pollen classification to enhance accuracy and efficiency. One such approach is the ‘Honey.AI’ system, which automates the scanning of honey sediment slides and uses AI algorithms to detect, identify and count individual pollen grains (https://honey-ai.com/es/home). Similarly, the ‘Classifynder’ system uses slide scanning technology to recognise and identify pollen grains from monofloral honeys (e.g., Manuka (*Leptospermum scoparium*) honey from New Zealand) on microscope slides, allowing semi-automatic and reproducible pollen classification^48,49^. However, automated microscopy still faces limitations, such as restricted throughput by slide scanning time and difficulties in segmenting overlapping pollen grains, which can affect identification accuracy^50,51^. In this context, recent studies have further demonstrated that deep learning models trained on annotated microscopic brightfield pollen image datasets (such as POLLEN24_SP, POLLEN73S or POLLEN23E) enable accurate and scalable classification of pollen grains from honey samples, although their performance remains strongly dependent on data quality and annotation accuracy^52–56^.

**Table 1:** A selection of recent literature for machine learning-based pollen recognition from microscopic brightfield images

Study	Number of classes/total no. of images	Architectures tested	Accuracy (%)
Valiente et al.^102^	16/19,000	VGG16, VGG19, InceptionV3, Xception, ResNet50, DenseNet201, MobileNetV2, EfficientNetV2M, PolleNetV1	95.7–98.0
Valiente et al.^56^	24/32,285	VGG16, VGG19, ResNet50, InceptionV3, Xception, DenseNet201, EfficientNetV2M and MobileNetV2, APFANet, PolleNetV2, PoleNetV2.mobile	96.3–98.0
Le et al.^66^	52/11,776	MobileNet, YOLOv11, Vision Transformer	22.6–62.4
Mahmood et al.^54^	116/7362	APFA-Net	97.2–98.3
Punyasena et al.^53^	46/ 14,153	ResNet34	82.3
Sevillano et al.^52^	46/ 19,500	Alexnet	97.9
Shamrat et al.^55^	46/ 19,612	PollenNet, AlexNet, MobileNetV2, VGG16, ResNet50	72.7–98.5
Zaykov^103^	5/data not provided	MobileNet, ResNet152, Xception, InceptionResNetV2, and VGG19	92.8–97.5

Multispectral imaging flow cytometry (MIFC), is a single-particle based high throughput method that in addition to brightfield images, generates fluorescence images of specific wave bands and scatter images of pollen^46,57–61^. Recent studies used MIFC images in combination with deep learning classifiers to identify fossil (with identification accuracy of 93% for 53 different pollen types)^62^ and modern zoophilous pollen (with identification accuracy of 96% for 35 different pollen types)^46,60^. However, no studies have yet compared how pollen images and features obtained from different pollen matrices, such as anthers, corbiculae or honey, impact the pollen classification from honey pollen. Generally, reference pollen images from anthers are easier to collect compared to pollen images from honey. Honey samples require more effort and carry the potential for erroneous labels of pollen image annotations from diverse samples, and therefore, it is essential to know if one can use anther pollen images to predict pollen in honey.

The MIFC method offers high reproducibility, as all pollen images are automatically saved, allowing for subsequent review and processing of the results. Feature analyses allow us to detect features that are difficult or even impossible for humans to visually analyse, and in the past, MIFC was already shown to be valuable for various pollen feature analysis^63–65^. Image features such as size, shape or fluorescence intensity can be automatically extracted from the image data and further used for pollen feature analysis and interpretation of classifier output, which is also known as the ‘Black box’ approach.

We aim to use MIFC images to classify pollen in honey by examining how the classification of pollen in honey is affected by pollen images obtained from different matrices that are used during the training process, including pollen from anthers and honey and to evaluate our results against traditional melissopalynological microscopy. In this study, we focus on six target (main) pollen, which we define as pollen types that are botanically characteristic of monofloral honeys and serve as key indicators for their classification, even if they occur in low relative abundance. We used images of different pollen matrices in our training data set to test for matrix effects of honey pollen classification, compared the agreement of different classifier types with traditional microscopy and explored, based on a set of human interpretable image features, which pollen features are most relevant for the discrimination of different pollen taxa.

## Results

In total, we trained the three different ML approaches with 1478 (out-of-domain configuration), 2378 (in-domain configuration), 3656 (mixed-domain configuration) and finally with 3756 (final configuration) pollen images. Across 13 monofloral honeys, pollen grains were assigned to one of the six target pollen, comprising 15,667 classified by bCNN, 29,384 by cCNN, and 17,545 by RF. In addition, 22,525 pollen grains were identified in 10 polyfloral honeys using the bCNN approach. The number of pollen grains per sample ranged from 158 (*Tilia*) to 3421 (*Castanea*) for monofloral honeys (Supplementary Table S9) and from 129 (summer blossom honey) to 7,734 (spring blossom honey) pollen grains for polyfloral honeys (Supplementary Table S9). MIFC measurement times varied usually between 1 and 10 min per sample for both monofloral and polyfloral honeys (Supplementary Table S9). On average, monofloral honeys contained 1205 pollen grains and polyfloral honeys 2451 pollen grains, with both having a mean measurement duration of 8 (monofloral) or 9 (polyfloral) min.

### Machine learning approach comparison for manually annotated target pollen

We tested three different machine learning experiments to determine which input is required for reliable pollen identification in honey samples (Table 2 and Supplementary Table S5–S7). In a first approach, we defined a set of a comparable number of ground truth images per main target pollen type and tested, which percentage of these ground truth images is correctly classified by the individual models. With that approach the bCNN in-domain brightfield configuration (pollen images extracted from honey in bright field mode) achieved the highest overall identification accuracy (95.1%), closely followed by the mixed-domain with all channels configuration (94.7%) (honey and anther pollen images in bright field and across nine fluorescence channels) (Table 2 and Supplementary Table 10). In contrast, the configurations trained exclusively on anther pollen used to classify the pollen from monofloral honey (out-of-domain brightfield: 79.8% and out-of-domain with all channels: 22.7%) yielded the lowest accuracies. Similarly, for both the cCNN and RF approaches, the in-domain brightfield configuration performed best (cCNN: 97.2%, RF: 90.8%), while the out-of-domain configuration with all channels achieved the lowest identification accuracy (cCNN: 41.4%, RF: 29.5%). Overall, the inclusion of fluorescence features during training with pollen images only from anthers appeared to substantially reduce identification accuracy (out-of-domain brightfield vs. out-of-domain with all channels), while adding fluorescence information to pollen in honey resulted in only minor changes (in-domain brightfield vs. in-domain with all channels). In the mixed pollen training, fluorescence even improved overall identification performance (mixed-domain brightfield vs. mixed-domain with all channels). However, configuration performance also varied by species. For example, the in-domain with all channels configuration slightly outperformed the mixed-domain with all channel configuration for *Brassica* pollen, whereas *Castanea* pollen was identified with high accuracy by nearly all configurations, except the out-of-domain with all channels. The mixed-domain with all channel configurations was considered the best configuration for further analyses. Out of the three classification approaches its accuracy in the bCNN approach differed by only 0.4% from the second best in-domain brightfield configuration, but achieved the highest performance in both the cCNN and RF approach (Table 2).

**Table 2:** Overview of the identification results for the target pollen classified from monofloral honeys with the six configurations using the built-in CNN ‘bCNN’ (left column), the customised CNN ‘cCNN’ (middle column) and the Random Forest ‘RF’ (right column) machine learning approaches

Configuration		Out-of-domain brightfield			Out-of-domain with all channels			In-domain brightfield			In-domain with all channels			Mixed-domain brightfield			Mixed-domain with all channels		
**Pollen matrix**		Anther pollen			Anther pollen			Honey pollen			Honey pollen			Anther & honey pollen			Anther & honey pollen		
**Channels**		2 (Brightfield)			12 (2 brightfield, 1 side scatter, 10 fluorescence)			2 (Brightfield)			12 (2 brightfield, 1 side scatter, 10 fluorescence)			2 (Brightfield)			12 (2 brightfield, 1 side scatter, 10 fluorescence)		
**Recall per pollen class** **(Individual honey samples summarised) in %**	target pollen	bCNN	cCNN	RF	bCNN	cCNN	RF	bCNN	cCNN	RF	bCNN	cCNN	RF	bCNN	cCNN	RF	bCNN	cCNN	RF
	*Brassica*	71.4	56.5	48.9	0.0	6.2	24.0	96.7	98.0	90.9	99.6	99.4	95.3	96.6	98.4	86.3	97.8	99.1	93.2
	*Castanea*	84.9	87.4	89.0	0.7	13.8	2.2	100	99.3	100	99.8	98.4	100	99.7	99.7	99.7	99.9	99.1	99.9
	*Centaurea*	50.3	50.0	27.6	99.4	72.2	64.6	84.7	75.2	61.8	86.0	96.8	88.6	82.2	77.8	58.0	92.4	96.8	89.9
	*Helianthus*	99.0	99.4	61.0	0.6	52.0	0.2	99.6	99.4	84.2	92.6	94.2	87.3	87.7	99.8	86.5	96.7	96.3	88.7
	*Phacelia*	92.0	91.9	38.7	14.8	59.5	52.2	95.9	90.2	90.7	88.4	95.5	85.8	99.1	91.1	91.7	96.6	93.8	92.9
	*Tilia*	81.3	73.7	74.0	21.1	44.8	34	93.8	85.0	82.4	90.6	95.8	81.8	89.3	88.6	84.9	84.6	98.3	80.5
**Averaged recall**		**79.8**	**76.5**	**56.5**	**22.7**	**41.4**	**29.5**	**95.1**	**91.2**	**85.0**	**92.8**	**96.7**	**89.8**	**92.4**	**92.6**	**84.5**	**94.7**	**97.2**	**90.8**

The total identification accuracy is marked in bold. The results for all pollen classes can be found in the appendix (Supplementary Table S10).

### Machine learning approach comparison for complete honey samples

**Fig. 1:**
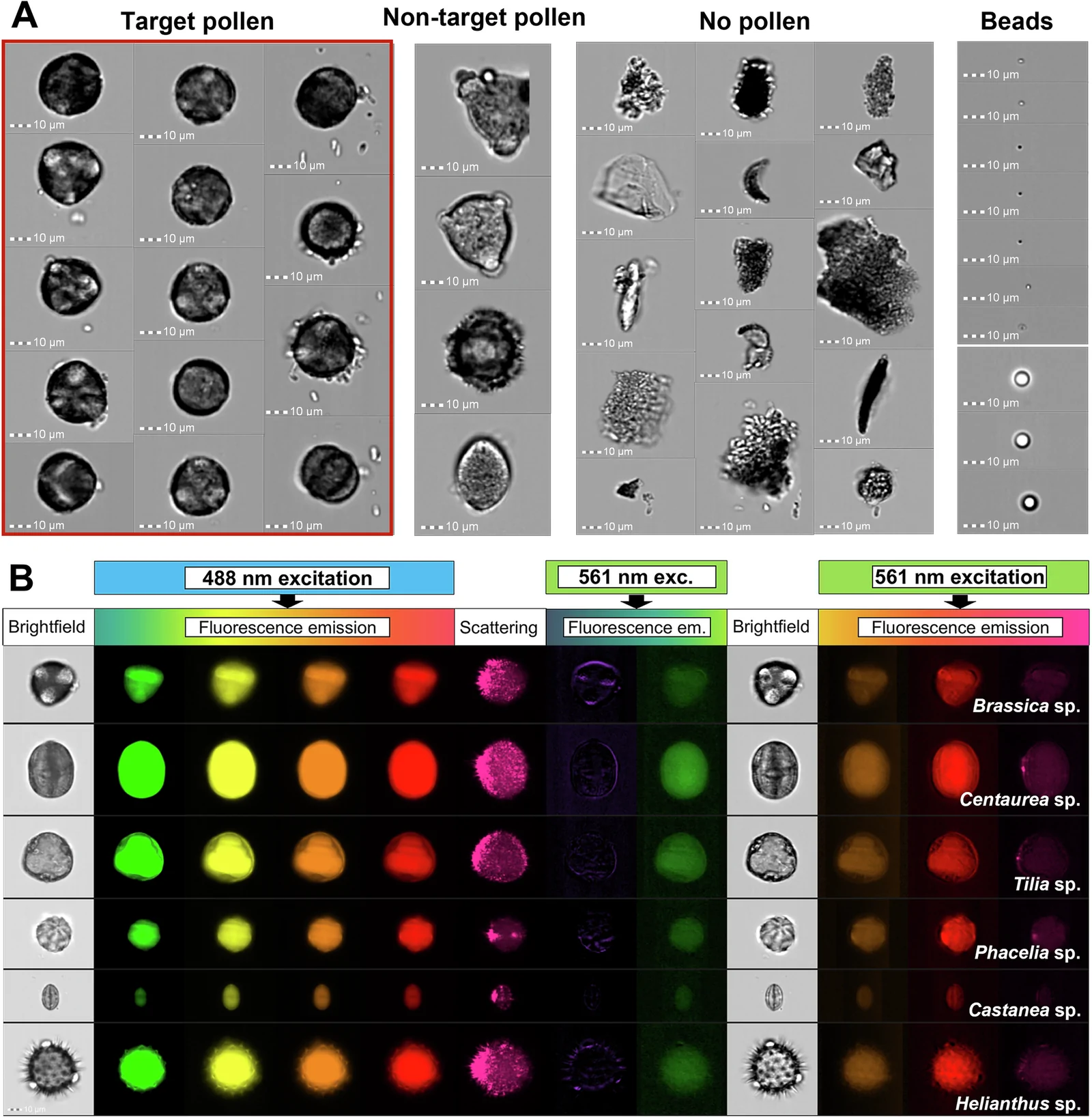
Exemplary images from a MIFC measurements. **A** Exemplary MIFC brightfield (Ch 01) images of a *Brassica* honey sample for target pollen, non-target pollen, no pollen and beads (calibration (top) and counting beads (bottom)). The basic machine learning configurations were only evaluated based on respective target pollen images (highlighted by a red box), while the final machine learning configuration was applied to all particle images of an individual measurement containing all of the shown classes. Some of the *Brassica* target pollen images may contain yeast contaminations (small particles attached or close to the pollen), representing a challenge for automated classification. **B** Exemplary MIFC multichannel images for all six investigated main pollen types (*Brassica* sp. type, *Castanea* sp. type, *Centaurea* sp. type, *Helianthus* sp. type, *Phacelia* sp. and *Tilia* sp. type).

**Fig. 2:**
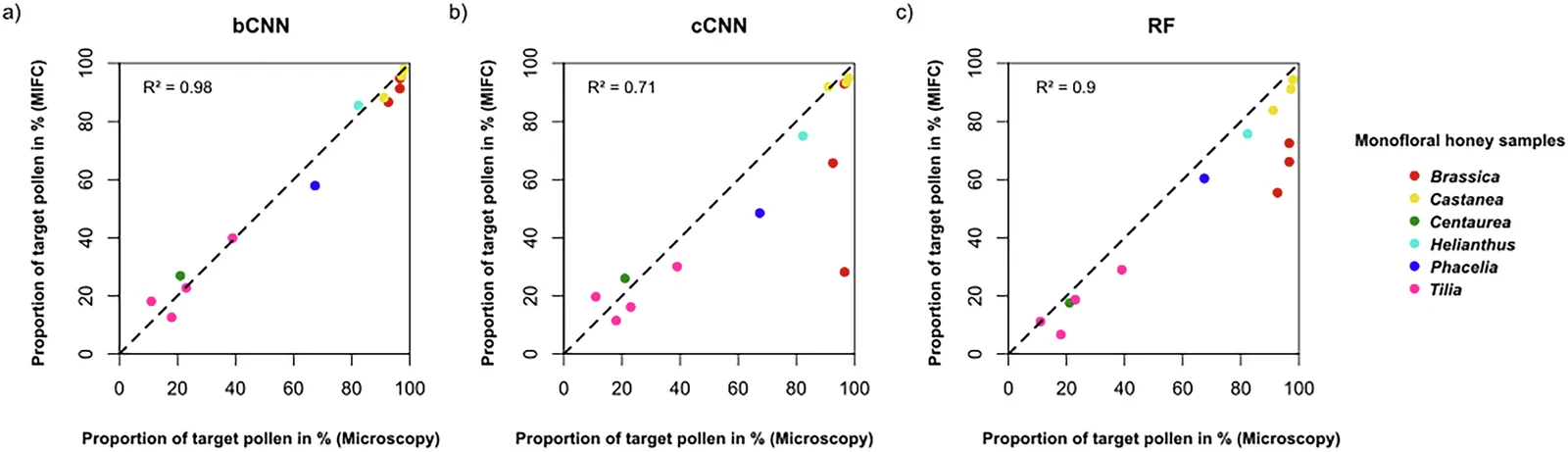
Comparison of microscopy and MIFC pollen proportions using bCNN, cCNN, and RF models. Scatter plots comparing microscopy and MIFC pollen proportion results from different monofloral honeys of **a)** the final bCNN configuration (based on the mixed-domain configuration with all channels), **b)** final cCNN configuration (based on the mixed-domain configuration with all channels), and **c)** the final RF configuration (based on the mixed-domain configuration with all channels). The six target monofloral pollen types are shown: *Brassica* (red), *Castanea* (yellow), *Centaurea* (green), *Helianthus* (turquoise), *Phacelia* (blue) and *Tilia* (pink). The dotted line indicates the 1:1 line for better visual comparison.

In a subsequent approach, the comparison of the monofloral MIFC-derived with the microscopy-derived target pollen type proportions was performed separately for each machine learning approach (Fig. 2, Supplementary Table S8, and Supplementary Fig. S1) using the respective final configuration based on the mixed-domain configuration with all channels and the second independent honey sample including all particles (Fig. 1, Supplementary Fig. S1). Among all three approaches, the MIFC results for bCNN show the closest agreement with the microscopy results (*R*² = 0.98), followed by the RF (*R*² = 0.90) and the cCNN (*R*² = 0.71) (Fig. 2). While the overall agreement between MIFC and microscopy is high, certain species, such as *Phacelia* and *Tilia*, show deviations between MIFC and microscopy results in the bCNN approach. In the cCNN approach, especially *Brassica*, *Phacelia* and *Tilia* proportions differ between MIFC and microscopy, whereas in the RF approach, *Brassica* and *Tilia* proportions show discrepancies.

**Fig. 3:**
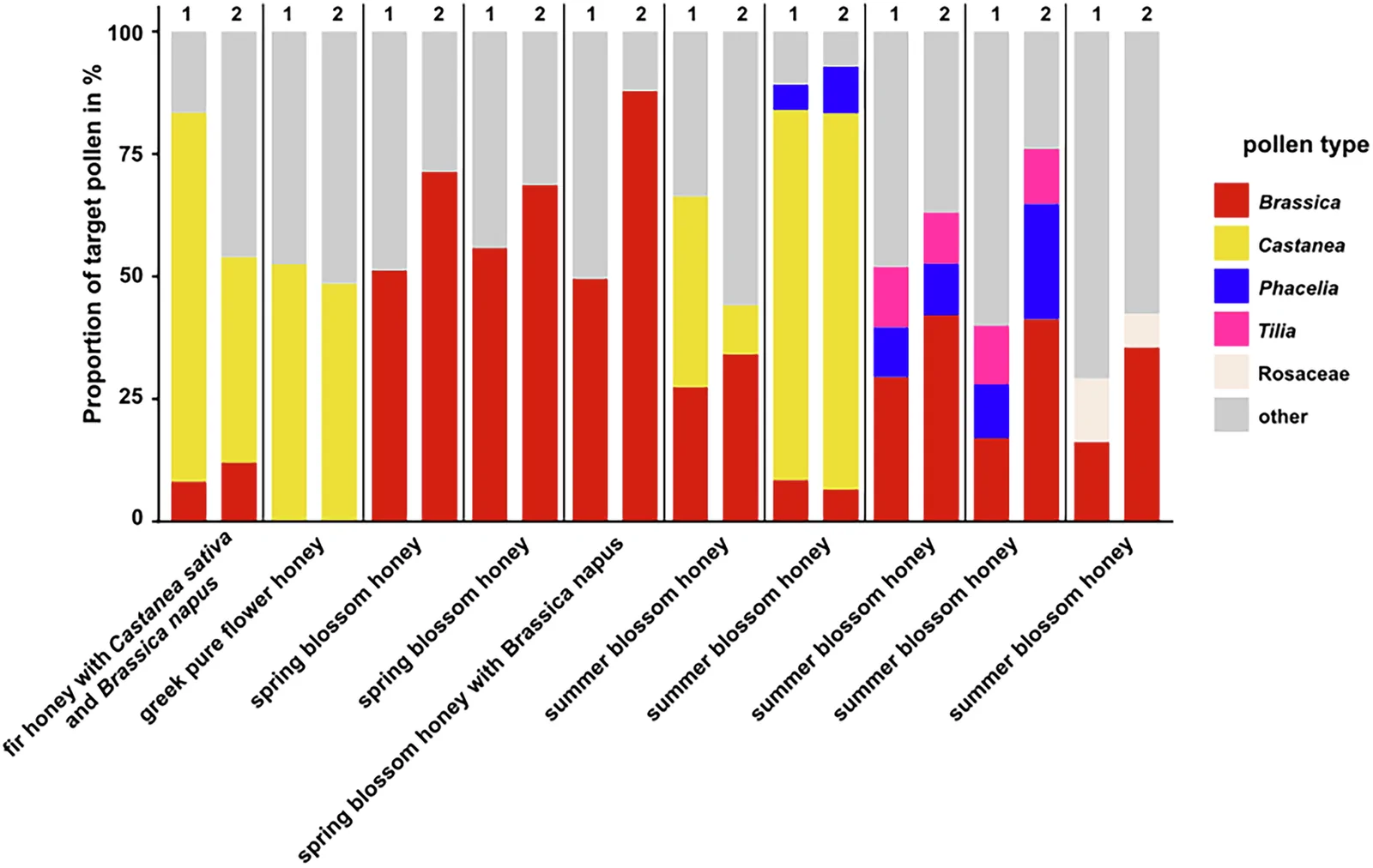
Target pollen proportions in polyfloral honeys determined by MIFC and microscopy. Stacked bar charts for the proportion of selected target pollen in polyfloral honeys obtained by MIFC (1) and microscopy (2) with the bCNN final configuration (based on the mixed-domain configuration with all channels). The target pollen types are shown: *Brassica* (red), *Castanea* (yellow), *Phacelia* (blue), *Tilia* (pink). In addition, the proportion of Rosacea (light pink) and other types (grey) of pollen is shown.

In the ten polyfloral honeys, we assessed which of the six target pollen were present and restricted the analysis to these detected taxa. The proportion of the detected pollen, determined using MIFC with the final configuration (based on the mixed-domain configuration with all channels), was compared with the proportion obtained by microscopy (Fig. 3 and Supplementary Table S11). Polyfloral honeys naturally vary considerably in their pollen composition, even if they are labelled as the same type, for example, as summer blossom honey. In most honeys, MIFC detected a lower proportion of *Brassica* and *Phacelia* pollen and a higher proportion of *Castanea* and Rosaceae pollen relative to microscopy. For *Tilia* pollen, both methods yielded similar values.

### Pollen image feature analysis

**Fig. 4:**
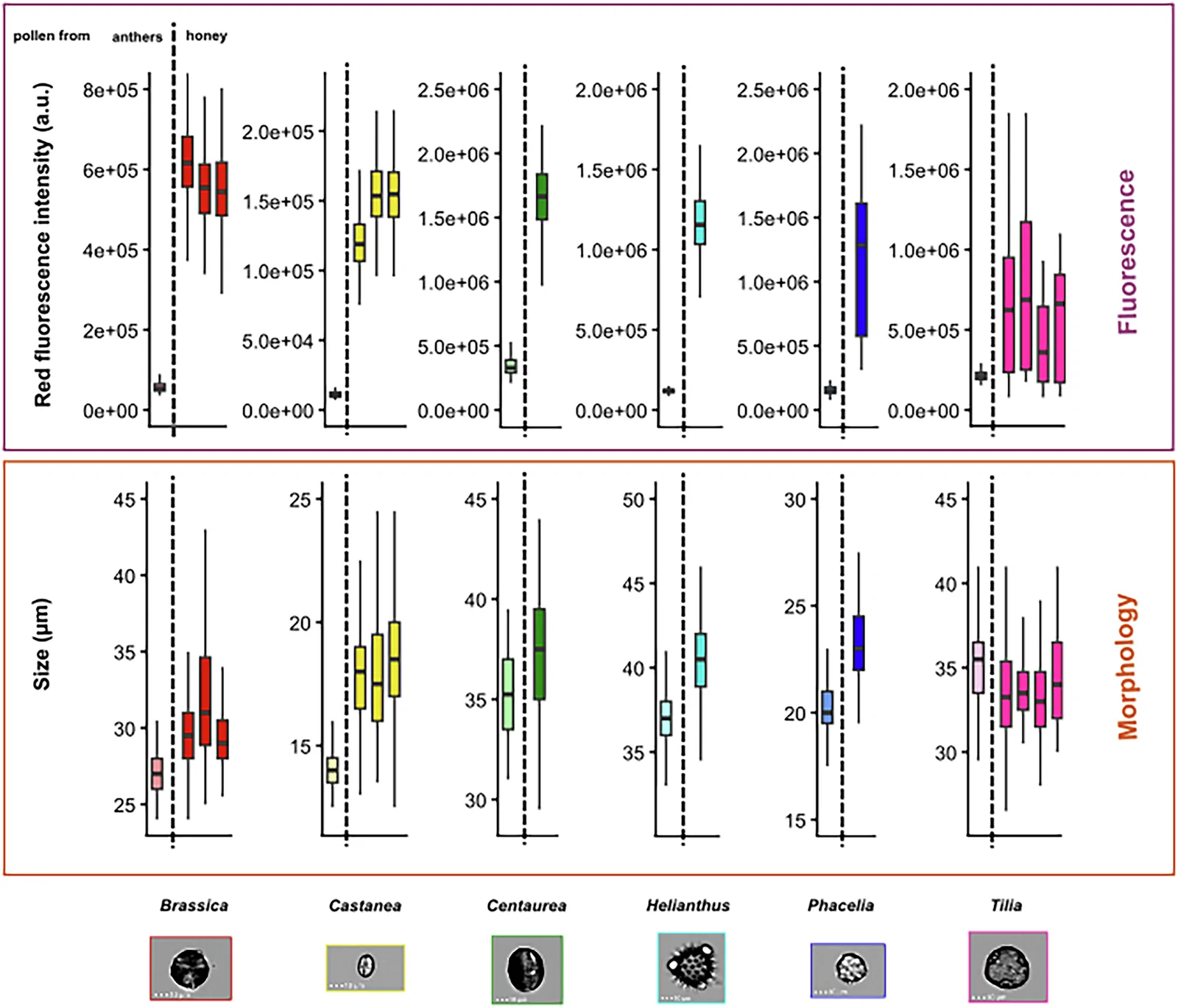
Distribution of pollen size and red fluorescence in anther and honey samples. Boxplots of the 19 individual anther and honey samples for the pollen features size and red fluorescence, representing values of all 200 and 100 pollen grains respectively measured. The six target pollen types are shown: *Brassica* (red), *Castanea* (yellow), *Centaurea* (green), *Helianthus* (turquoise), *Phacelia* (blue) and *Tilia* (pink). Light colours (left boxes) indicate pollen from anthers, whereas dark colours (right boxes) represent pollen from honey; the respective boxplots are additionally separated by a dashed line. The displayed features are: above - red fluorescence intensity (Ch 5) as fluorescence feature; below - length together as size/morphology feature. Microscopic images of pollen from honey from the six target pollen types, captured using MIFC, are shown in brightfield below the boxplots.

For a better understanding of the low performance of out-of-domain model configurations (Table 2), we exemplarily tested whether pollen collected from anthers and honey differ in two image features using pollen size and red fluorescence across 19 anther and honey training samples of the target pollen (Fig. 4). Overall, *Brassica*, *Castanea*, *Centaurea* and *Helianthus* pollen in honey showed much higher red fluorescence intensities than the respective anther pollen while pollen size was more comparable between anther and honey samples. Across species, the coefficient of variation (CV) differed between pollen from anthers and honey. For size, pollen grains from honey generally showed higher relative variability, with CV ratios ranging from 1.3-fold (*Tilia*) to 3.5-fold (*Brassica*) compared to anther pollen (Supplementary Table S12). In contrast, fluorescence variability showed species-specific patterns. Pollen from honey exhibited higher CVs in *Helianthus*, *Phacelia*, and *Tilia* (2.0-, 2.0- and 5.3-fold, respectively), whereas pollen from anthers showed higher fluorescence variability in *Brassica* (5.2-fold). For *Castanea* and *Centaurea*, fluorescence variability was comparable between pollen from honey and anthers (CV ratios close to 1) (Supplementary Table S12).

**Fig. 5:**
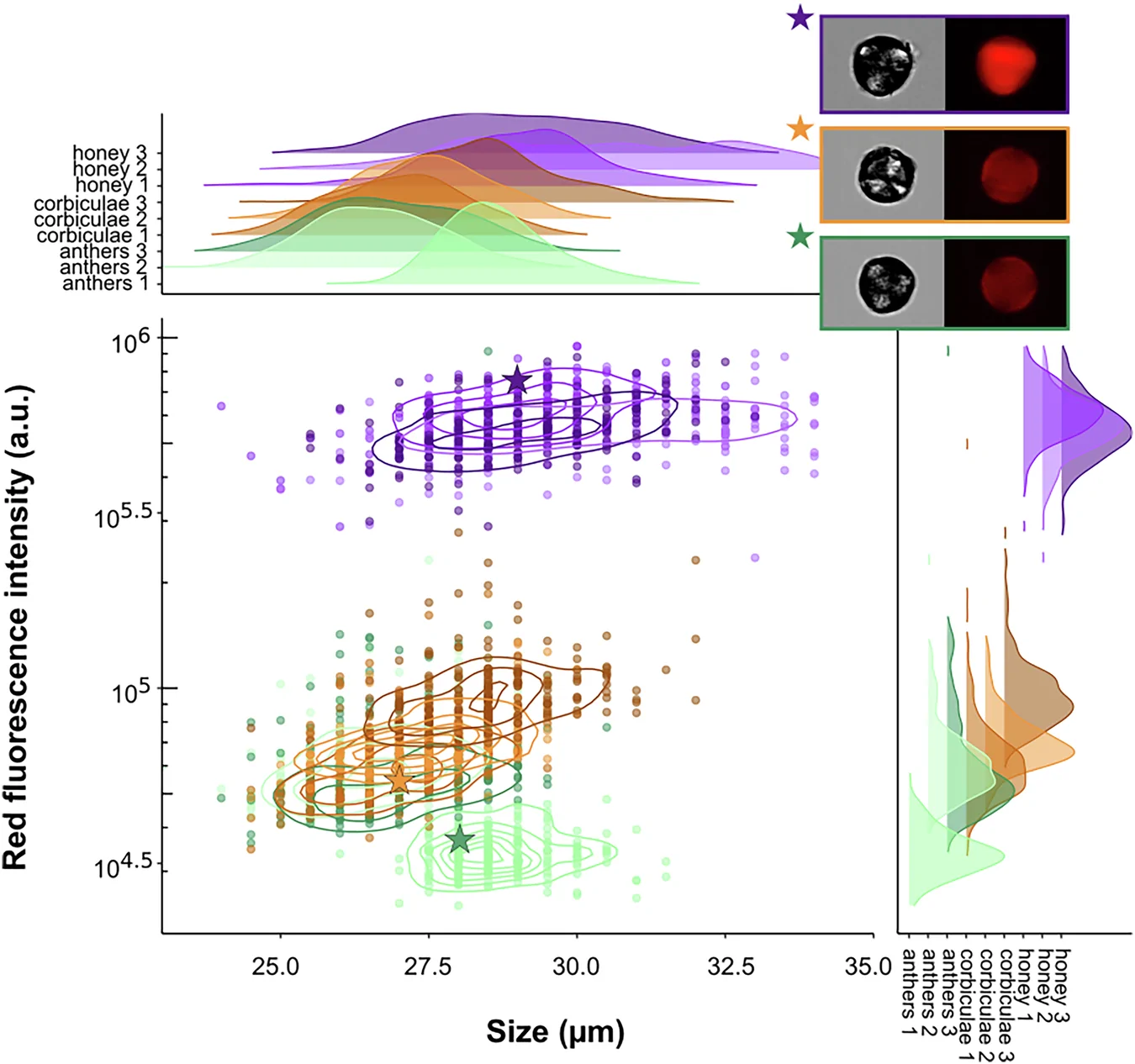
Density and dot plot for two selected *Brassica* pollen features from different pollen sources. The features shown are pollen size (length) and red fluorescence intensity (intensity Ch 5). The pollen grains were taken from three different plant/anther (green tones), three different corbiculae (orange/brown tones) and three different honey (purple tones) samples. Exemplary microscopic images of pollen from various sources, captured by MIFC, are displayed in bright field and red fluorescence channels, which are marked with a star on the diagram.

While *Brassica* pollen from anthers (mean: 27.4 µm ± 1.43 SD), corbiculae (mean: 27.6 µm ± 1.29 SD), and pollen extracted from honey (mean: 29.4 µm ± 1.87 SD) did not differ significantly in pollen size (*p* > 0.05, Supplementary Table S13), red fluorescence intensity, interestingly, allowed a clear distinction between pollen from honey and pollen from anthers or corbiculae (*p* < 0.001, Fig. 5, Supplementary Table S13). *Brassica* pollen from honey exhibited a 7–10 times higher red fluorescence (mean: 576,767 a.u. ± 107,679 SD) compared to anther (mean: 54,353 a.u. ± 41,398 SD) and corbicula (mean: 78,552 a.u. ± 31,125 SD) pollen. These patterns can also be found in *Castanea*, which was one additional exemplary pollen type for which sufficient data were available in this context (Supplementary Fig. S2).

The feature comparisons shown in Figs. 4 and 5 do not adequately explain the differences in class-accuracies in Table 2 for out-of-domain configurations with fluorescence channels included. This suggests that model performance may depend more on the between-class-differences than on differences in honey and anther pollen alone. Therefore, we next examined between-class differences within in-domain honey samples.

**Fig. 6:**
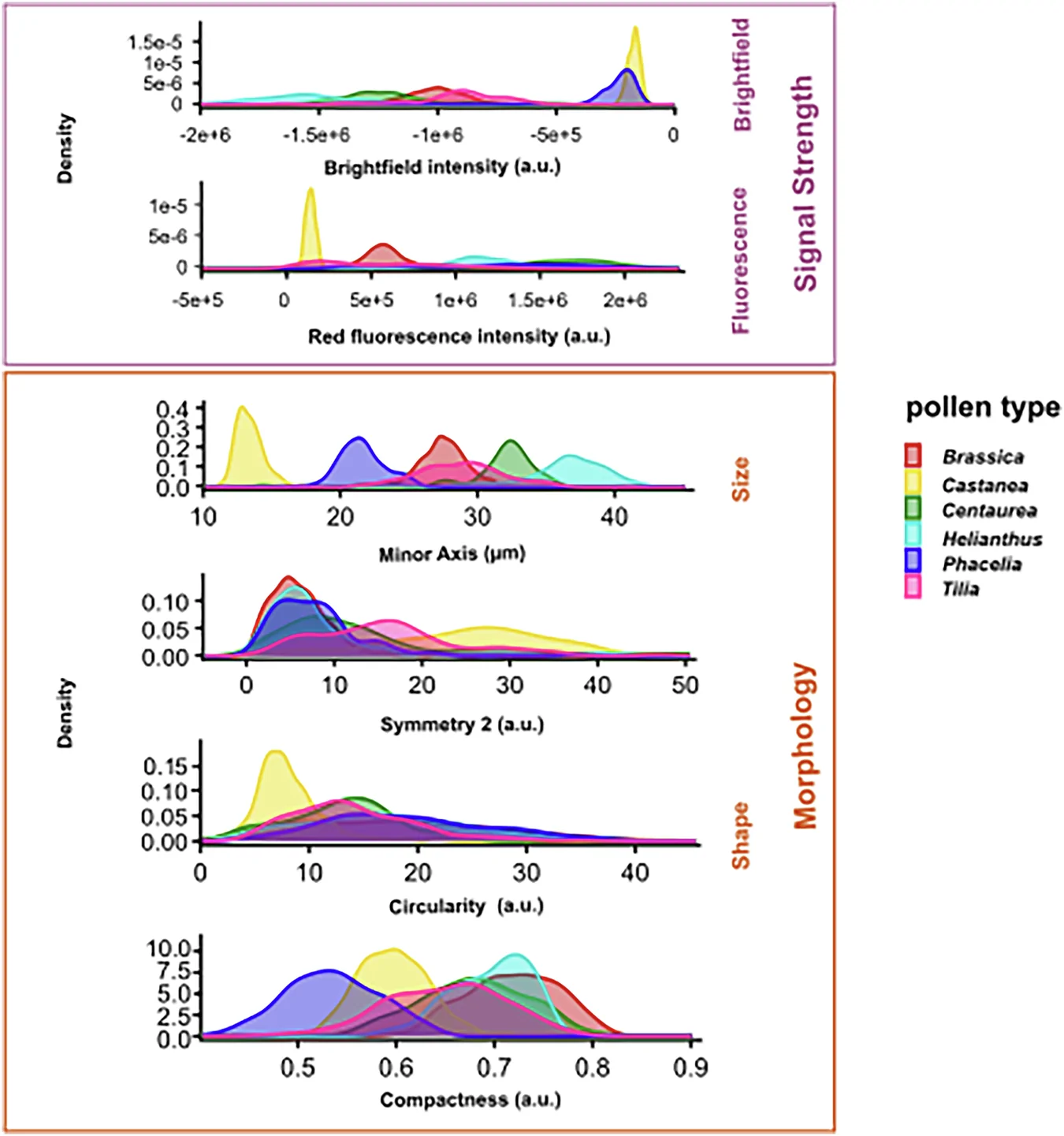
Density plots of the combined honey pollen samples for six pollen features used for the random forest training. The six target pollen types are shown: *Brassica* (red), *Castanea* (yellow), *Centaurea* (green), *Helianthus* (turquoise), *Phacelia* (blue) and *Tilia* (pink). The displayed features include signal strength features: brightfield intensity (Ch 1) and red fluorescence intensity (Ch 5) and morphology features: minor axis, symmetry 2, circularity and compactness.

Analysis of the six most important features of pollen from honey based on the mixed-domain configuration with all channels from the RF approach revealed two signal strength features (brightfield and red fluorescence intensity) and four morphological features (size: minor axis and symmetry; shape: circularity and compactness) (Fig. 6). Based on brightfield intensity, *Phacelia* and *Castanea* can be distinguished from each other and from the other pollen types. However, differentiating the remaining species is more challenging due to their similar brightfield characteristics. Red fluorescence further enables discrimination among *Brassica*, *Castanea* and *Tilia*. Among the morphological features, the minor axis proved to be the most informative, allowing separation of all species except *Tilia*. Symmetry 2 contributed little to differentiation, while circularity was only useful for distinguishing *Castanea* and *Centaurea*. Compactness, in turn, was suitable for separating *Castanea* or *Phacelia* from the other species. In general, the plant species can be distinguished primarily by their pollen size, followed by their fluorescence features.

**Fig. 7:**
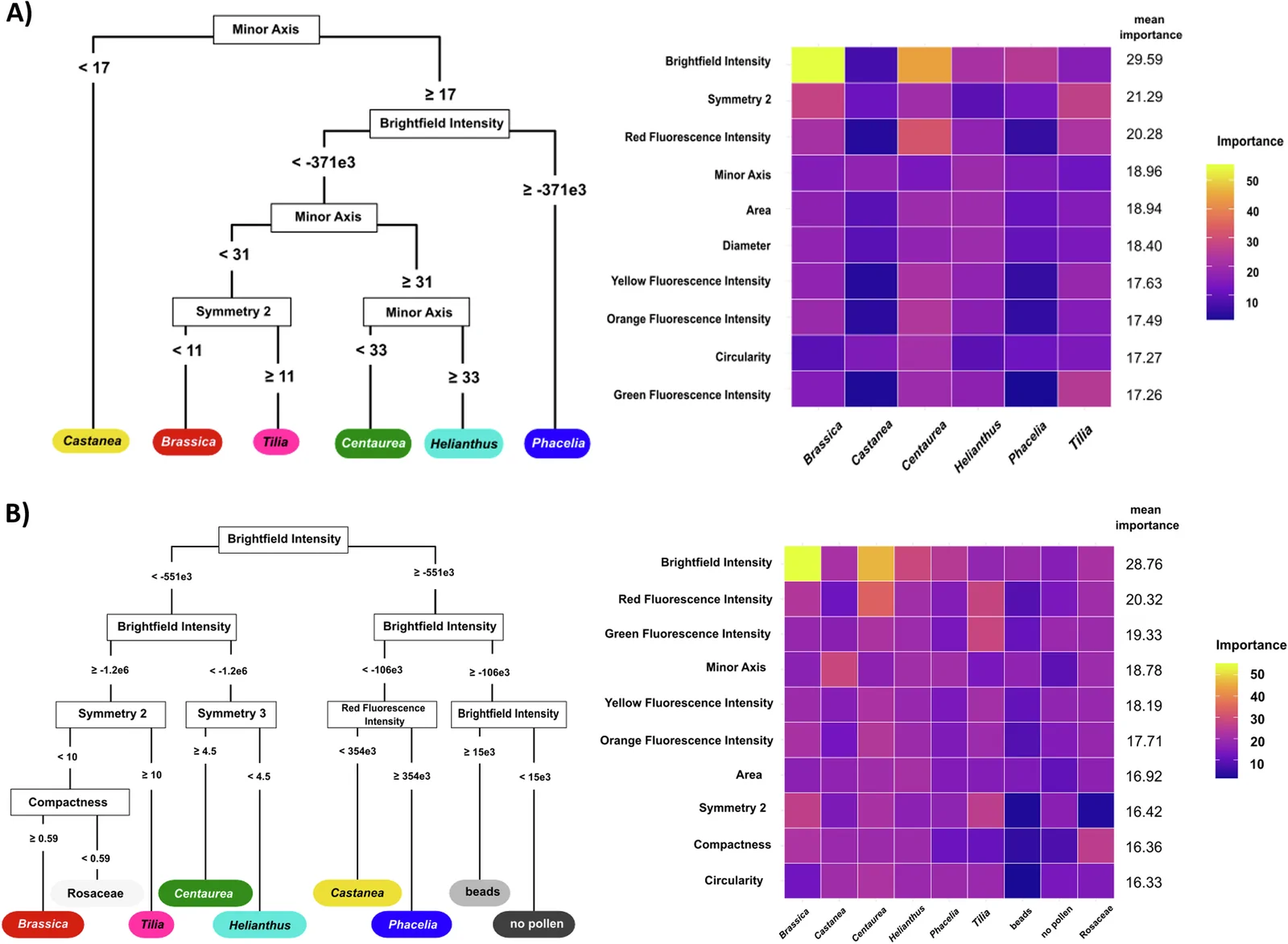
Exemplary random forest decision tree and variable importance for pollen classification. left: Decision tree illustrating the pollen features for all six target pollen classes (**A**) and for all six target pollen classes including the ‘beads’ (grey), ‘no pollen’ (black) and ‘Rosaceae’ (light grey) class (**B**) in honey samples based on the final configuration of the RF approach. The six target pollen types are shown: *Castanea* (yellow), Brassica (red), *Helianthus* (turquoise), *Centaurea* (green), *Tilia* (pink) and *Phacelia* (blue). Right: Variable importance of the 10 most important features based on the final configuration of the RF approach, ordered by global impact (highest at the top).

To better understand underlying decision mechanisms of the CNN models, we also explored the feature-based machine learning technique of decision trees and random forests for only the target pollen (Fig. 7a) and for all classes in the training (Fig. 7b). According to the RF with the final configuration, the features ‘Brightfield Intensity’, ‘Symmetry 2’ and ‘Red fluorescence intensity’ showed the highest discriminatory power. Brightfield Intensity and Symmetry 2 were, in part, also comparable to how a human taxonomist would discriminate between different pollen types. Brightfield Intensity indicates how much light passes through a particle. In pollen classification, this intensity can be used as a distinguishing feature between bright, less optically dense pollen (e.g. *Phacelia*) and dark, optically dense pollen types (e.g. *Brassica*) (Fig. 4). Symmetry 2 is a shape feature that describes the tendency of the pollen grain to have a single axis of elongation and therefore two lobes. Red fluorescence intensity in pollen reflects the emission of fluorescent signals from pigments and cell wall components such as phenolic compounds, which emit light in the red spectral range when excited by the cytometry laser. Most interestingly, the relevant features in the decision tree with only the target pollen differed substantially from those of the decision tree including all classes. When focusing exclusively on the target pollen, size features were largely sufficient for differentiation (Fig. 7a). However, signal intensity and shape features were more relevant when all classes needed to be distinguished from each other (Fig. 7b). The parameter importance of the RF trained only on pollen classes revealed that size-related and a few signal features were higher ranked. In contrast, the RF, including all particle classes, shifted towards a stronger dependence on multiple fluorescence signals alongside size, reflecting the greater complexity of distinguishing pollen from non-pollen particles (Fig. 7).

## Discussion

An automated high-throughput method for identifying pollen in honey can reduce manual effort, inconsistencies and costs for analyses and by that overcomes limitations in traditional pollen analyses. Therefore, the main objective of this study was to identify the best way of quantifying target pollen proportions in honey by means of MIFC. Since anther samples are the easiest way to create an extensive image reference database, these were the initially preferred source material to create an AI-based classification tool. However, we found strong feature deviations in pollen from honey compared to pollen from anthers or corbiculae, which makes robust automated detection difficult when only anther pollen images are used to classify pollen in honey. Incorporating both brightfield and fluorescence images and features from mixed samples containing both anther and honey pollen resulted in the highest prediction accuracy for unseen honey pollen samples. Among the three tested machine learning approaches (bCNN, cCNN, RF), the cytometer’s built-in module (bCNNs) achieved the best identification accuracy compared to microscopic counts for unseen mono- and polyfloral honey samples.

Many previous studies have used machine learning approaches to identify pollen on microscopic images with classification accuracies between 68%–96%^21,49,60,66–68^. These studies demonstrate that accuracies exceeding 90% can be achieved under controlled or idealised conditions, where image quality is high and morphological variation is limited. However, the majority of these approaches were evaluated exclusively under in-domain conditions, meaning that training and evaluation were restricted to the same sample type. For example, Chica (2012)^67^ evaluated their approach exclusively on bright-field microscopic images of bee pollen loads, whereas Pospiech et al. (2021)^21^ trained and tested their models solely on pollen images extracted from honey samples. Likewise, Gimenez et al. (2024)^68^ reported detection performance under in-domain conditions using microscopic images of pollen embedded in complex environmental samples. In contrast, Le et al. (2025)^66^ introduced a domain shift by training their model on anther pollen images and predicting pollen from honey samples. Their approach resulted in variable classification accuracies (70.21%–92.47%) depending on the honey type, which is roughly comparable to that of our out-of-domain brightfield configuration (76.5%–79.8%). However, fluorescence properties were not considered by Le et al.^66^. A more distinct performance decrease under domain shift was reported by Lagerstrom et al.^49^, who transferred models trained on reference pollen images (from the Australian National University’s pollen reference collection) to real honey samples and observed a substantial decrease in accuracy from 95%–96% to 68%–81%. Consistent with this observation, all three of our machine learning approaches with the mixed-domain configuration using all channels reached accuracies above 90% (bCNN: 94.7%, cCNN: 97.2%, RF: 90.8%) in the monofloral honeys containing only annotated target pollen. However, identification accuracy typically declines in different honey types or environmental samples, where increased morphological variability, presence of debris and image artefacts introduce additional sources of error^69^. We observed the same pattern in our data, with a decrease in classification performance from annotated pollen to entire monofloral and further polyfloral honey samples, reflecting domain shifts and increased variability of pollen features between these matrices. This is consistent with previous findings, highlighting the strong dependence of model performance on image quality and the challenges posed by real-world datasets for both classical machine-learning and deep-learning approaches^69^. It should also be noted that the differences between microscopic and MIFC main target pollen type proportions also arise from the different amounts of totally considered pollen. Taken together, these results underline the importance of training strategies and reference data that account for matrix-specific variability when applying image-based classifiers to complex honey samples.

Random forests (RF) and convolutional neural networks (CNN) differ in how they learn and represent information. RFs operate on predefined, manually extracted features and build an ensemble of decision trees that learn relationships within these structured features. Their performance, therefore, depends strongly on the quality and completeness of the features^70^. CNNs, by contrast, learn hierarchical feature representations directly from raw images, allowing them to capture complex spatial patterns and subtle morphological differences without manual feature design. This ability is particularly advantageous when image variation is high, as is the case with pollen from different matrices and images^70^. In our study, the RF approach achieved generally the lowest accuracy among the three approaches, most likely because the predefined feature set did not fully represent the morphological and fluorescence variability present in the data. The CNN-based approaches learned these informative patterns more effectively and generally outperform classical approaches when datasets exhibit high morphological diversity, although they remain sensitive to natural image variation and artefacts^69^. Both CNNs achieved similar accuracies for monofloral honeys containing only annotated target pollen. The cCNN model was trained using a 95% training split of the dataset, with evaluation performed on a 5% holdout set, which constrained both the training data volume and the final accuracy achieved. Besides restrictions of the training datasets, numerous cCNN optimisation opportunities could yield results that would surpass the bCNN’s limited controllability and performance capabilities. Furthermore, the cCNN can be exported and shared openly in accordance with FAIR data principles, while also allowing flexible model adjustments, while a bCNN can be created without much prior ML knowledge. But for the purpose of the ML comparison, allocating additional computational resources would have created an inequitable comparison.

The comparison between in-domain and out-of-domain configurations further highlights a clear domain shift across all approaches (RF, bCNN and cCNN). The results also demonstrate that anther-based training data alone do not contain the full information required for accurate classification of pollen from honey due to domain shift effects. The mixed domain results confirm that providing all multispectral image channels from different pollen matrices (anther and honey sample origin) provides highest accuracies. We can assume that both the different image origin as well as the image properties (brightfield and fluorescence) contain complementary information, resulting in a high accuracy performance of our final configurations (90.9%–97.2%). This pattern was consistent across the three machine learning approaches we evaluated. Pollen identification improves whenever models were trained with brightfield and fluorescence images from both anthers and honey samples, demonstrating that the mixed-domain configuration captures the relevant morphological and spectral variation more comprehensively than single-domain setups. Identification accuracy, however, also depends on the specific pollen type. *Brassica* pollen, for example, can be identified slightly better when the training is based solely on pollen from honey, although the difference in the identification accuracy is neglectable. *Helianthus* and *Phacelia* pollen, in turn, were already identified with a high accuracy using the out-of-domain configuration. While for *Castanea* pollen almost all configurations are equally suitable.

Taxonomists work primarily with the morphological features of pollen, such as size, shape, polarity, or apertures^19^. In theory, good pollen type discrimination should be achieved when features can be identified that show large enough inter-specific and ideally small intra-specific differences. In addition to sufficient inter-specific variation, low intra-specific variability is important for good discrimination because high within-type variation increases the likelihood of overlap between feature distributions of different pollen types. When pollen feature variability of the same pollen type is large, consistent classification becomes difficult, and misclassifications become more likely. Low intra-specific variability, therefore, helps to form more distinct feature clusters that improve discrimination performance.

For the RF model discriminating the six pollen types, pollen size emerged as the primary determinant in MIFC-based classification (Fig. 6). The strong impact of ‘Minor axis’ shows that size-related features capture substantial inter-specific variation, whereas shape features such as ‘Symmetry’ or ‘Circularity’ contribute comparatively little, likely due to higher intra-specific variability. The importance of ‘Brightfield intensity’ and ‘Red fluorescence intensity’ demonstrates that signal strengths complement morphological information, particularly for taxa with overlapping size ranges. Nevertheless, the limited separability of *Tilia* highlights that some pollen types share highly similar morphological features and signal strengths, which limits the classification performance regardless of the selected features. Overall, these findings suggest that reliable pollen type discrimination relies mainly on a combination of size and spectral features, while shape-based features play only a minor role.

When the RF focused solely on the target pollen, size-related features were largely sufficient for separation, reflecting clear morphological differences within this subset (Fig. 7a). However, when all particle classes from the training data were included, the RF shifted towards a stronger dependence on signal and shape features. This change indicates that, in a more heterogeneous dataset, size alone cannot ensure adequate separation and must be complemented by structural and signal information. The substantial differences between the features selected by the two decision trees further emphasise that the underlying decision mechanisms vary depending on whether the classifier must distinguish among morphologically similar pollen types only or additionally differentiate pollen from non-pollen particles. A key challenge in pollen recognition for complete MIFC measurements is precisely this discrimination of pollen from ‘no-pollen’ particles. The fraction of ‘no pollen’ included a broad range of particles and was quite arbitrary, depending on the individual samples. From the different RF approaches, we see that depending on the no-pollen classes, the features with the highest discriminatory power can shift strongly, so the selection of a representative ‘no pollen’ class is important. The RF trained on pollen only versus the RF trained on all classes demonstrates that the composition of the no-pollen classes strongly affected which features showed the highest discriminatory power. This means that the decision behaviour of the RF is not fixed but can shift considerably depending on which classes are included in the model. To obtain a more robust representation, we tried to include ‘no pollen’ particles from several samples, which, based on the comparative microscopy results, proved effective for full-sample classification of the selected honeys. In contrast to the RF model, the two bCNN and cCNN-based model results (including all MIFC channels and pollen from anthers and honey) were most consistent with microscopic results, which can be explained by the far larger amount of discriminatory power of a CNN in comparison to a Random Forest with 20 features.

For the investigated target pollen types, it turned out that especially red fluorescence differed significantly between the pollen from anthers/corbiculae and honey. These strong differences might be the main reason why out-of-domain configuration trained with only anther pollen and including fluorescence channels showed such a low performance (22.7%–41.4%) when the model was used to classify pollen of the target pollen types collected from honey samples. In pollen from anthers, fluorescence mainly originates from phenolic compounds, flavonoids, carotenoids and proteins embedded in the sporoderm^71,72^. Until now, the underlying mechanisms of the observed up to 7–10-times increase in pollen fluorescence when stored in honey are still unclear. One possible explanation might be a different water content of pollen from anthers compared to pollen in honey, potentially changing morphological and fluorescence signals. Although this explanation seems unlikely since all pollen MIFC measurements are performed in D-PBS^72^, Pöhlker et al. (2013)  demonstrated that the state of hydration has a significant impact on the autofluorescence spectra of pollen, with dry grains exhibiting significantly weaker and spectrally shifted emissions compared to hydrated grains. In honey, the typical water content of around 17%–18% prevents complete dehydration of the pollen and in addition, pollen grains are embedded in a viscous but hygroscopic matrix rich in sugars, amino acids, and antioxidants^73,74^. This assumption is supported by the comparison with the corbiculae pollen. Anther pollen is not processed and exhibits the lowest fluorescence intensity. Corbiculae pollen, on the other hand, consists of anther pollen collected by honey bees and mixed with low proportions of nectar, which contains among others various sugars^75^. The fluorescence intensity of corbiculae pollen is slightly but not significantly higher than that of pollen from anther, but much lower than that of pollen from honey, which remains in the sugar rich solution for a longer time. Honey consists of approximately 80% various sugars (e.g. fructose (38%), glucose (31%), maltose (3.4%)^76^, turanose (1.7%)^77^ and other multiple sugars^78^). The intense further processing (e.g. enzymatic digestion) by honey bees and the high sugar concentration might finally lead to the observed increase in pollen fluorescence.

However, despite the good overall agreement of microscopy and MIFC, especially *Brassica* pollen was underestimated by the cCNN approach. By searching for reasons explaining these results, we recognised that in some honeys, *Brassica* pollen showed several attachments (Fig. 1), which were identified as yeast cells. Yeasts frequently occur in nectar and honey, mainly introduced by pollinators during their flower visits and can adhere to pollen grains^79^. In the training dataset, such pollen was partially included so far, but the results highlight that in future, the training dataset should include a wider range of representative pollen images.

In this study, the training dataset consisted of a relatively small number of images (3756 in total) for the final configuration, yet this configuration achieved robust performance. The small dataset size mainly arose from the target of a balanced dataset and the necessary effort to manually annotate pollen images from honey samples, e.g. in honey of *Tilia* and *Centaurea* the number of pollen per sample was between 100–178 images. The consistent results despite the small dataset highlight the greater potential of this approach as the dataset expands. An extended reference pollen database could further enable the detection of honey adulteration. Those configurations, including pollen images from anther and honey samples perform best indicates that anther samples can be added to the training dataset, reducing the need for more labour-intensive annotation of honey samples. So far, the present study has demonstrated the reliable recognition of target pollen in monofloral honeys. For *Brassica* honeys, the variation between MIFC measurements was low, with a coefficient of variation of 4.48%. This degree of variation is comparable to the repeatability reported for microscopy, which has a repeatability limit of 5.19% when counting 1000 pollen grains^80^ (DIN 10760). This suggests that the reproducibility of MIFC for *Brassica* is well within the range of established microscopic precision.

The comparison of MIFC-based and microscopic pollen proportions in the polyfloral honeys reveals both the potential and limitations of image-based cytometry for complex samples. Because the established classifier recognises only six pollen types included in the training data so far, only polyfloral honeys were selected, which, based on microscopic melissopalynological analyses, contain at least one of the six main pollen types, and the analysis was restricted to these target pollen types only. The natural compositional variability of polyfloral honeys, even within the same label, may explain method discrepancies. Classifiers trained with MIFC images consistently underestimated *Brassica* and *Phacelia* while overestimating *Castanea* and Rosaceae relative to microscopy, potentially arising from deviations in morphological or signal features. The smaller grains such as *Brassica* and *Phacelia* may be more challenging for the final configuration to separate from the heterogeneous background fraction, whereas *Castanea* and Rosaceae exhibit clearer structural or signal features that facilitate their recognition. The similar values obtained for *Tilia* for both methods were unexpected, as the feature analyses indicated that *Tilia* pollen does not have any clear morphological or signal features that distinguish it significantly from other target taxa. This consistency might therefore be explained by the generally low frequency of *Tilia* in these honeys rather than by a particularly high detectability of this pollen type. So far, no other pollen types were currently included in the classifier to focus on matrix and classifier aspects as a first step. Definitively, the main aim is to develop classifiers with a much broader application range. In that step, it would also be relevant to test pollen type classes which are known in microscopic melissopalynological studies to be challenging to classify (e.g. pollen types of the family Brassicaceae, Rosaceae or Asteraceae or between Brassicaceae and Oleaceae).

However, the botanic origin of honey can still not solely be identified based on any image-based method as there is a non-linear correlation between the pollen number of a certain taxa and the definition of the botanic origin. It is important to note that to finally provide information about the latter, other analyses need to be performed^1,7,8,18^.

Considering the comparable results alongside its short analysis times (1–10 min), MIFC represents a powerful new screening tool for monofloral honey samples. The longer duration required for classical microscopy, often 30–60 min per sample^2^, emphasises the practical advantage of this approach. The ability of MIFC to deliver consistent measurements within minutes, regardless of sample complexity, underscores its potential for high-throughput workflows and routine quality assessment. Its capacity to store image data further enables reproducibility and future re-evaluation with improved classifiers. Inter-laboratory comparisons between taxonomist and MIFC measurements will be an important next step to assess the robustness of pollen analyses across laboratories. In this study, a comparably low number of samples was analysed considering the high measuring capacity of MIFC. However, we wanted to carefully consider a limited set of samples in detail, but a higher number of samples should be investigated in further studies.

Conclusively, our results demonstrate that as a proof-of-principle, MIFC combined with machine learning provides a fast, automated and high-throughput method for identifying target pollen in monofloral honeys. This approach can effectively complement conventional microscopy by enabling rapid preliminary screening of monofloral honey samples using MIFC. For polyfloral honeys, the method allows the identification of predefined target taxa, although performance is currently constrained by the limited number of pollen types included in the reference dataset. Nonetheless, the obtained results indicate that MIFC can capture meaningful patterns in complex honey matrices and that further expansion of the training data will enhance its applicability to polyfloral compositions. As classifiers will improve, this framework provides long-term flexibility for quality assessment, authenticity testing and broader monitoring of botanical composition. In future, the approach should be tested using a wider variety of mono- and polyfloral honeys in combination with a more comprehensive training database.

## Methods

### Pollen samples from anthers

Pollen from anthers was obtained from different projects, but all followed the same pollen collection and extraction protocols presented by Dunker et al.^60^ and Hornick et al.^64,81^ (Supplementary Table S1). Sampling took place from 2021 to 2023 in Saxony and Saxony-Anhalt (Germany).

### Corbiculae pollen samples

Four corbiculae pollen samples from *Brassica napus* (*n* = 3) and *Castanea sativa* (*n* = 1) were analysed solely for the purpose of comparing pollen features from different matrices. All corbiculae pollen samples were provided by the University of Hohenheim—State Institute of Bee Research, which also carried out the microscopic pollen analysis. Samples were collected in 2019 from honey bee colonies in Baden–Wuerttemberg, Germany, using common pollen collection traps.

### Honey samples

Here, 13 monofloral honeys were analysed, each containing one of the following target (main) pollen: *Brassica napus* type (*n* = 3), *Castanea sativa* (*n* = 3), *Centaurea cyanus* (*n* = 1), *Helianthus annuus* type (*n* = 1), *Phacelia tanacetifolia* (*n* = 1) and *Tilia* sp. (*n* = 4). The monofloral honey samples were provided either by the University of Hohenheim - State Institute of Bee Research or the Lower Saxony State Office for Consumer Protection and Food Safety LAVES—Institute of Apiculture in Celle (Supplementary Table S1). In addition, 10 polyfloral honeys with the following labels were analysed: summer blossom honey (*n* = 2), summer blossom honey with *Brassica napus* (*n* = 1), spring blossom honey (*n* = 5), fir honey with *Castanea sativa*, *Brassica napus* and Rosaceae pollen types (*n* = 1) and Greek pure flower honey (*n* = 1). The polyfloral honey samples were provided either by local beekeepers, the German Beekeepers’ Association (DIB e.V.), the Lower Saxony State Office for Consumer Protection and Food Safety LAVES—Institute of Apiculture in Celle or beekeepers from Greece (Supplementary Table S2). Microscopic pollen analyses are available for all honey samples (Supplementary Tables S1 and S2). As it is common practice in melissopalynological analyses, pollen abundance data were available as proportions per pollen type. That is why we compared our MIFC pollen type proportions with the ones determined by microscopy.

### Sample preparation

All pollen samples from anthers and homogenised corbiculae pollen were prepared according to Dunker et al.^60^. Briefly summarised, Pollen Isolation Buffer (PIB, modified from Aloisi et al.^82^) was added to the samples, followed by vortexing and 5 minutes of sonication to separate aggregated grains. The samples were then filtered, using size-appropriate filters (30, 50 or 70 µm) for anther pollen and a 100 µm filter for corbiculae pollen (CellTrics, Sysmex, Norderstedt, Germany), centrifuged at 4000 × *g* and the resulting pellet was resuspended in Dulbecco’s phosphate-buffered saline (D-PBS).

All honey samples were prepared according to DIN 10760 until the decanting step. In brief, 10 g of each honey sample was weighed in a 50 ml centrifuge tube (LABSOLUTE®, TH Geyer, Germany), then 20 ml of distilled water was added to dissolve the honey. The mixture was centrifuged for 10 min at 1000 *g* (Heraeus Megafuge 16R centrifuge, ThermoFisher Scientific, Osterode, Germany). The supernatant liquid was poured off and another 20 ml of distilled water was added to completely dissolve the sugar crystals in the honey. The solution was then centrifuged again for 5 min at 1000 × *g* and the supernatant liquid was decanted once more. Subsequently, 1 ml of D-PBS (Dulbecco’s phosphate-buffered saline (without calcium and magnesium), Biowest, Nuaillé, France) was added to the centrifuge tube and mixed thoroughly. The honey sample was then pipetted into a 1.5 ml reaction tube, including a filtration step with a 100 µm filter (CellTrics, Sysmex, Norderstedt, Germany) and centrifuged for 2 min at 4000 × *g*. Finally, the supernatant was removed, and 70 µl D-PBS was added.

### Multispectral imaging flow cytometry (MIFC)

The multispectral imaging flow cytometer Imagestream X Mk II was used for all pollen measurements (Amnis, part of Cytek, Amsterdam, Netherlands). In order to ensure reproducible data acquisition, the instrument was calibrated on a daily basis using Speed Bead Calibration Reagent® (Cytek Biosciences, Seattle, USA). Each sample (pollen from anthers, corbiculae and honey) was measured at a 40x objective (numeric aperture 0.75, resolution 0.5 µm/px, field of view 60 × 128 µm) and connected to an autosampler according to Dunker et al.^60^. The cytometer utilised three lasers: 488 nm laser with 5 mW intensity, 561 nm laser with 20 mW intensity and 785 nm laser with 0.1 mW intensity and two independent CCD cameras. As brightfield channels, Ch1 and Ch9 were used, as well as Ch6 as scatter channel. The fluorescence channels are defined by the following excitation and emission wavelengths Ch 2 (528/65 nm BP filter), Ch 3 (577/35 nm BP filter), Ch 4 (610/30 nm BP filter), Ch 5 (702/85 nm BP filter), Ch 7 (457/45 nm BP filter), Ch 8 (537/65 nm BP filter), Ch 10 (610/30 nm BP filter), Ch 11 (702/85 nm BP filter) and Ch 12 (762/35 nm BP filter). An image of a representative pollen grains, shown in all available channels, is included in Fig. 1B.

Counting beads (ThermoFisher Scientific, Eugene, USA), a standardised suspension of microspheres that are brightly fluorescent, were added to anther, corbiculae and honey samples before the measurement as an internal standard for fluorescence measurements. Data collection was terminated when either 5000 particles had been measured or 10 min had passed. For each anther sample, one cytometric measurement was performed and used to create the model training data. For each honey sample, two immediate independent cytometric measurements were performed. These two measurements represent technical replicates: the first was used to generate the training data, while the second measurement was used for classification with the resulting configuration. When more than one honey sample exists for a given target pollen, as in the case of *Brassica*, *Castanea* and *Tilia*, these distinct samples represent biological replicates. However, the honeys containing *Phacelia* and *Tilia* pollen were measured a third time, as the first two measurements contained only a small amount of pollen. This was expected, as these honey types usually contain naturally low pollen amounts. The measurements of the *Phacelia* and *Tilia* honey samples were then merged into one measurement for training and one measurement for classification.

### Image extraction and annotation

Sharp images of single pollen grains from anthers, corbiculae and honey were isolated from debris using a bivariate plot of brightfield channel intensities in the IDEAS Software (Version 6.2.187.0, Amnis, part of Cytek, Amsterdam, Netherlands), as described by Hornick et al.^64^. These images were further refined by excluding those containing multiple pollen grains or additional debris particles, based on particle size criteria. To ensure high data quality for the training data (pollen from anthers and honey), all remaining particle images were manually annotated and divided into the categories: ‘target pollen’, ‘non-target pollen’, ‘no pollen’ (e.g., debris) and ‘beads’ (including speed and calibration beads). This annotated dataset was used for training. For classification, the annotated pollen from honey data was initially divided into the same categories, with classification focused on identifying the ‘target pollen’ class (Fig. 1A). With the final configuration, the full unannotated measurement was classified for comparison with the microscopic results (Fig. 1A). Exemplary multichannel images for each of the six main target pollen types are shown in Fig. 1B. For the analysis of pollen features, only images with single, sharp, centred and debris-free pollen grains, produced with this method, were used.

### Feature selection and extraction

The analysis focused on 20 pollen features selected based on the findings of Dunker et al.^60^ and their transferability to classic microscopic pollen analysis. To also assess the value of fluorescence properties for automated classification, fluorescence features were included alongside morphology-related features. The selected features comprised Area, Circularity, Compactness, Diameter, Elongatedness, Height, Brightfield Intensity (Ch 1), Green Fluorescence Intensity (Ch 2), Yellow Fluorescence Intensity (Ch 3), Orange Fluorescence Intensity (Ch 4), Red Fluorescence Intensity (Ch 5), Scatter Intensity (Ch 6), Length, Major Axis, Minor Axis, Perimeter, Symmetry 2, Symmetry 3, Symmetry 4 and Width. These features cover two categories: morphology, representing shape and size, and signal strength representing brightfield, scatter and fluorescence signal intensities (Supplementary Table S3). For the comparison of pollen features among the different matrices, the analysis was restricted to one size-related feature (‘Length’) and one fluorescence feature (‘Red Fluorescence Intensity’ (Ch 5)) as an indicator for pigment compounds. In the following, the term ‘Size’ refers specifically to the feature ‘Length’ as according to Hornick et al.^64^, ‘Length’ was identified as the most suitable feature to represent pollen size of differently shaped and oriented pollen. All feature calculations were performed using an ‘AdaptiveErode mask’, which ensures an optimal fit for each pollen grain by applying a threshold value of 95%^64^. The ‘AdaptiveErode mask’ detects pixels surrounding the particle so that they touch the input boundary at a defined minimum radius threshold and subsequently erodes 5% of the particle’s boundary (IDEAS 6.2 User’s Manual, Amnis).

### Machine learning

To determine the optimal architectures and input parameters for pollen in honey classification using MIFC, two methodological approaches were used: an image-based approach using convolutional neural networks (CNN) and a feature-based approach using random forests (RF). We choose CNNs for pollen identification because they learn discriminative pollen features directly from the images and reliably capture even small differences. Their robustness to natural variations and strong performance even with limited training data make them ideal for this task. In addition, Random Forest (RF) is a feature-based machine learning approach that builds an ensemble of decision trees, offers high robustness against overfitting and provides direct insight into feature importance. We additionally used RFs because they perform well on structured feature sets and provide a complementary, interpretable baseline to validate and compare the CNN results. For the first CNN approach, the built-in Amnis AI module CNN (Version 2.0.7) (bCNN) of the imaging flow cytometer was used. This integrated software enables the direct classification of MIFC image data without intermediate processing steps, but offers only limited possibilities for customisation. In the second CNN approach, the experiment was conducted using a customised CNN (cCNN), which allowed for greater flexibility in adjusting training parameters and settings. In a third approach, a random forest (RF) was created.

### Machine learning configurations

We designed our machine learning configurations not only to observe how accurately these algorithms could classify pollen particles from MIFC, but also to gain knowledge on the usability of pollen features based solely on material from anthers and honey. Anther reference pollen images are easier and less erroneous to collect and it could be assumed that pollen in honey show different morphological and fluorescence properties, but we do not know how much information this accounts for. Therefore, we designed six configurations (Table 3). First of all, we wanted to observe how much of the necessary information for accurately classifying samples is shared across the data of anthers and honey. We consider this the out-of-domain configuration. Our target (in-)domain was pollen from honey in all configurations, but here we trained on the anther particle images (out-of-) domain. As the in-domain configuration, on the other hand, we consider training on honey, which is also considered the easiest setting because all pollen images are expected to be most comparable. Finally, we refer to mixed-domain configurations if we train both on honey and anther pollen images. Here, we intended to observe whether the out-of-domain data aids in accurate classification by adding important information. The six configurations also allow us to separate the contributions of shape, size, texture, and fluorescence.

**Table 3:** Overview of the training and classification data used in the different configurations

Configuration	Training data				Pollen classes	Classification data
	Anther pollen	Honey pollen	Brightfield images	Fluorescence images		
Out-of-domain brightfield	x	-	x	-	Target pollen, ‘beads’, ‘no pollen’	Manually annotated target honey pollen
Out-of-domain with all channels	x	-	x	x		
In-domain brightfield	-	x	x	-		
In-domain with all channels	-	x	x	x		
Mixed-domain brightfield	x	x	x	-		
Mixed-domain with all channels	x	x	x	x		
Final (based on mixed-domain with all channels)	x	x	x	x	Target pollen, ‘beads’, ‘no pollen’ ‘Rosaceae’	Complete honey sample

We trained three approaches (bCNN, cCNN and RF) with the six different configurations (out-of-domain, in-domain, mixed-domain) (Table 3). By intention, we did not further optimise the cCNN classifier to compare results based on comparable hardware requirements and invested time for optimisation.

The entire training was based on the annotated pollen images or their feature values collected from anther and honey samples with the following twelve channels: Ch 1 + Ch 9 (brightfield), Ch 2–5 + Ch 7–8 & 10–12 (fluorescence) and Ch 6 (scatter). A balanced dataset containing 200 pollen images per class was used for training. Exceptions occurred when fewer pollen grains were available. For *Centaurea*, pollen images in honey were difficult to detect, thus for anther pollen only 100 images of grains could be used, for *Tilia* anther pollen 178 grains and for the honey pollen classes 100 (*Centaurea*) and 178 (*Tilia*) images were included, respectively. An overview of all honey and anther pollen samples including the training parameters can be found in the appendix (Supplementary Table S1). The following classes were used across all trainings: *Brassica*, *Castanea*, *Centaurea*, *Helianthus*, *Phacelia*, *Tilia*, ‘beads’, ‘no pollen’ (anything other than pollen and beads, e.g. debris). In addition to single *Castanea* pollen grains, the *Castanea* pollen class also included 100 images containing multiple *Castanea* pollen grains, as these occurred more frequently in the samples. Approximately 60% of particles representing non-target particles were dirt, calibration and counting beads and pollen from non-target plants. The first classification was carried out only for the annotated target pollen images from the second independent measurement of the honey samples (Fig. 1).

Based on the six configurations tested across the three approaches, we selected the best-performing configuration and used it as the basis for training one final, extended configuration to enable comparison with the microscopy results. The final configuration for all approaches was trained using the annotated target pollen classes together with ‘no pollen’, ‘beads’ and the additional pollen class ‘Rosaceae’ (Table 3), comprising various pollen from plants of the Rosaceae family. The ‘Rosaceae’ class was added because the microscopic honey analysis for *Centaurea* and *Tilia* honey revealed a high relative abundance of this pollen type, which had previously caused misclassification when applied to the complete sample images. All preprocessing and training steps up to this point remained unchanged. The classification in this case was carried out on the complete unseen second honey sample measurement.

### Deep learning—built-in CNN (bCNN)

The first approach was carried out using the built-in AI software module *Amnis AI* (Version 2.0.7, Amnis part of Cytek). The Amnis AI model uses a Keras Application Programming Interface (API) version 2.1.5 with the TensorFlow version 1.7.0 library and a VGG16 architecture to train seven CNN configurations^83^ (Table 3). In this approach, the taxa-annotated image data from the imaging flow cytometer served directly as input for training and classification without any further preprocessing steps.

### Deep learning—customised CNN (cCNN)

For the second approach, we conducted an extensive search for a suitable CNN architecture and hyperparameters for augmentation and training.

#### Preprocessing

Since the MIFC image export process is error-prone, extensive error-correction preprocessing was needed. Exporting MIFC images from multiple files automatically is not supported by the MIFC software (IDEAS, Version 6.2.187.0, Amnis part of Cytek), and custom libraries had to be used (https://www.openmicroscopy.org/bio-formats/); this necessitated automatically detecting data type errors, clipping image values, and removing defective pixel values.

We optimised a selection of techniques for preprocessing. First, we removed the background bias. Here, the mean value per channel was calculated on the background, and the value was subtracted from the particle image, foreground and background. Second, we masked the particles’ backgrounds with the given particle masks; third, we normalised the images channel-wise using statistics (mean and standard deviation) from the training dataset; and fourth, we normalised the images channel-wise per image (instance norm).

#### Models

Since we were free in model selection, we used different models with varying depths and architectures for optimisation. We organised our model selection into three groups to cover a broad spectrum of modern computer vision architectures when doing a model architecture search: We began with a series of smaller and less complex architectures. Here, we included ResNet-18 and ResNet-25^84^ as lighter baseline models to establish performance benchmarks with fewer parameters and computational requirements. Since we expected the latest CNN architectures to perform better, we extended our search to another group of CNN architectures. This group comprised deeper and more sophisticated convolutional architectures: ResNet-50^84^, Wide ResNet-50 and Wide ResNet-101^85^, ResNeXt-50 32x4d and ResNeXt-101 32x8d^86^, and EfficientNet-V2-M^87^. These models represent significant advances in CNN design through increased width, cardinality, and compound scaling strategies. As the most state-of-the-art group, we extended our search to Vision Transformer models ViT-B/16 and ViT-B/32^88^ as pure transformer-based approaches, as well as ConvNeXt-Small and ConvNeXt-Base^89^, which modernise CNNs using design principles inspired by transformers.

#### Augmentation

For augmentation, we optimised the use of several transformation techniques tailored to the specific characteristics of our multi-spectral pollen dataset. Unlike typical RGB imagery, our multi-spectral data precluded the use of colour-based augmentations such as colour jitter or solarisation, as these would corrupt the meaningful spectral information across channels. However, several geometric and mixing-based augmentations proved particularly suitable for our pollen classification task: Random mirroring and rotation are valid transformations because pollen grains are approximately spherical and exhibit rotational symmetry, making orientation-invariant features desirable. Random resized cropping addresses the natural size variability of pollen particles, which appear at different scales due to both inherent biological size differences between species and varying distances to the camera, as well as developmental stages (mature versus immature grains) and hydration states (de-hydrated versus hydrated pollen). The cropping operation additionally forces the network to learn from partial views of pollen grains, improving robustness to occlusions and edge cases in real-world samples. We also employed mixup^90^ and cutmix^91^, which are particularly valuable for distinguishing between morphologically similar pollen taxa, as these methods encourage the model to learn more discriminative features by training on interpolated examples. Random erase^92^ enhances generalisation by simulating missing information and forcing the network to learn from fewer visual cues, which is crucial given the limited availability of labelled pollen samples. Finally, we applied synaptic scaling^93^ as an effective regularisation technique that stabilises training dynamics and prevents overfitting in deep networks. For each augmentation method, we tested default parameters against bypassing them to determine their individual contributions to classification performance.

#### Experimentation

Deep learning model performance is highly sensitive to hyperparameter choices, particularly for specialised tasks like multi-spectral pollen classification, where optimal configurations are not established in the literature. We therefore conducted systematic hyperparameter optimisation to ensure fair comparison across architectures and maximise classification performance before evaluation.

#### Hyperparameter search

The hyperparameter search was conducted utilising the ASHA (Asynchronous Successive Halving Algorithm), which evaluates not only the final accuracy but also implicitly rates better fast converging and fast inference methods, implemented via the Ray Tune PyTorch library. Based on the ASHA-Algorithm, the optimal combinations depend on the accuracy and convergence behaviour, and no model received all the budget needed to fully and perfectly tune it. The result, therefore, is not the optimal-accuracy combination but the optimal achievable combination under these specific time and computational limitations. The optimisation process involved a budget of 2500 runs and was performed on a subset of the mixed domain configuration training dataset, utilising 5% for training and 1% for testing. The search space and the optimal hyperparameters found are described in the Appendix and were evaluated based ASHA-Algorithm (Supplementary Table S4).

The optimal model we found was Wide ResNet-50-2. We trained it with a batch size of 128 for 100 epochs. Data was loaded from a zipped dataset as open microscopy TIFF images interpreted as int32. Optimisation used AdamW with a learning rate of 0.003, momentum 0.9, weight decay 0.005 and synaptic scaling loss rate of 0.033. Regularisation included MixUp (*α* = 0.45), CutMix (*α* = 0.4), and Random Erase (0.05). Learning rate scheduling used Cosine Annealing, with a 5-epoch linear warm-up and a minimum LR of 0.0. Image preprocessing applied bilinear interpolation, train/val cropping to 288px, and validation resize to 320px. The model operated in AMP (automatic mixed precision) mode and trained with instance normalisation enabled (Supplementary Table S4).

#### Evaluation

We trained the models for the six different configurations, utilising 95% of the respective training sets, evaluating the best training epoch with a 5% validation set. The final model performance was evaluated on the test sets for each configuration. Note that for optimal accuracy, one could employ cross-validation with multiple validation splits and ensemble methods trained on the full training data; however, computational constraints limited our approach to single validation splits per configuration.

### Feature-based learning—Random forest (RF)

The third machine learning approach was conducted in R (v.4.3.0^94^) using the ‘*randomForest*’ package^95^. The training was performed on the 20 previously selected numerical pollen features, with pollen classes as response variable. The random forest consisted of 500 decision trees. To determine which features, contribute most to distinguish between pollen classes, a variable importance analysis was performed using all 20 features. The importance values were based on the ‘MeanDecreaseAccuracy’, which quantifies how much the overall accuracy of the model decreases when the values of a particular feature are randomly permuted. To illustrate the structure of the training data, two different decision trees were extracted and visualised from the random forest. One tree contained all pollen classes including ‘beads’, ‘no pollen’ and ‘Rosaceae’ and one tree contained solely the target pollen classes. Both trees were trained on the eight most important features using identical R code with tuning parameters explicitly set using the ‘*rpart’* package (cp = 0.01, minsplit = 100).

### Statistical analysis

Statistical analyses and data visualisation were carried out using R (v.4.3.0)^94^ and, among others, the following packages: ‘*emmeans*’^96^, ‘*caret*’^97^, ‘*randomForest*’^95^, ‘*rpart*’^98^, ‘*ggplot2*’^99^, ‘*scales*’^100^ and ‘*rpart.plot*’^101^.

To determine which of the six configurations was best suited for identifying pollen grains in honey, the overall-accuracy was considered, where classified pollen classes were compared with the actual classes for each configuration (Supplementary Tables S5–S7). Classifications assigned to ‘no pollen’ or ‘beads’ were not considered in the evaluation, which ensured that only pollen classes were assessed. To evaluate the results of the final configuration in relation to the microscopy results, classified and true pollen classes were assessed as described above and the percentage of correct classifications was calculated (Supplementary Table S8). For this evaluation, particles identified as ‘no pollen’, ‘beads’ or ‘Rosaceae’ were not taken into account so that the analysis focused on the relevant pollen classes.

To test whether pollen from different source materials differed in size and red fluorescence intensity, we performed an Analysis of Variance (ANOVA) followed by a Tukey post hoc test for pairwise comparison. Assumptions for ANOVA were checked prior to analysis by testing residuals for normality using the Shapiro-Wilk test and homogeneity of variances using Levene’s test. The formula for the ANOVA was structured as follows: feature_mean ~ matrix, where feature_mean is the size or fluorescence median data (grouped by individual sample) and matrix was anthers, corbiculae or honey.

For the analysis of pollen size and fluorescence of all 19 anther and honey samples, the data on fluorescence intensity and length were aggregated depending on the number of individual samples per species in order to obtain robust representative values per species. For species with only a single sample, the median per measurement was calculated. For species with more than one sample, the median of each individual sample was first calculated, and then the mean of these medians was used as the final value. The same procedure was applied separately for fluorescence intensity and pollen size, with only data from honey samples included in the analysis.

## Supplementary information

Supplementary material_final_REVISION

## Data Availability

The datasets generated and analysed during the current study are available in the zenodo-repository (https://zenodo.org/records/18174733).

## Code availability

The code for generating the results analysed during the current study are available in the zenodo-repository (https://zenodo.org/records/18174733).
