# Supplementary material for: Profiling of pollen in honey using multispectral imaging flow cytometry
Source: NPJ Sci Food. 2026 Oct 30;10:337. doi: 10.1038/s41538-026-01188-6 (PMC13722696; doi:10.1038/s41538-026-01188-6)
Supplement: Supplementary file 1 [file 41538_2026_1188_MOESM1_ESM.docx]

**Supplements**

**Supplementary Tables**

**Supplementary Table S1**: Overview of all anther and honey samples, including origin and number of pollen images used for the identification of pollen in monofloral honeys.

|  | **class** | **sample type** | **provided from project (sample location)** | **microscopic pollen analysis carried out by** | **image no. in training** |
| --- | --- | --- | --- | --- | --- |
|  | *Brassica* | anther | NutriBee (Leipzig) | - | 200 |
|  | *Brassica* | honey | University of Hohenheim - State Institute of Bee Research | University of Hohenheim - State Institute of Bee Research | 200 |
|  | *Brassica* | honey | University of Hohenheim - State Institute of Bee Research | University of Hohenheim - State Institute of Bee Research | 200 |
|  | *Brassica* | honey | University of Hohenheim - State Institute of Bee Research | University of Hohenheim - State Institute of Bee Research | 200 |
|  | *Castanea* | anther | PolDiv (Leipzig) | - | 200 |
|  | *Castanea*  training | honey | University of Hohenheim - State Institute of Bee Research | University of Hohenheim - State Institute of Bee Research | 300 |
|  | *Castanea* | honey | University of Hohenheim - State Institute of Bee Research | University of Hohenheim - State Institute of Bee Research | 200 |
|  | *Castanea* | honey | University of Hohenheim - State Institute of Bee Research | University of Hohenheim - State Institute of Bee Research | 200 |
|  | *Centaurea* | anther | iNTERACT (Tilkerode) | - | 100 |
|  | *Centaurea* | honey | Lower Saxony State Office for Consumer Protection and Food Safety LAVES - Institute of Apiculture | Lower Saxony State Office for Consumer Protection and Food Safety LAVES - Institute of Apiculture | 100 |
|  | *Helianthus* | anther | PolDiv (Leipzig) | - | 200 |
|  | *Helianthus* | honey | University of Hohenheim - State Institute of Bee Research | University of Hohenheim - State Institute of Bee Research | 200 |
|  | *Phacelia* | anther | iNTERACT (Schortewitz) | - | 200 |
|  | *Phacelia* | honey | University of Hohenheim - State Institute of Bee Research | University of Hohenheim - State Institute of Bee Research | 200 |
|  | *Tilia* | anther | PolDiv (Leipzig) | - | 178 |
|  | *Tilia* | honey | Lower Saxony State Office for Consumer Protection and Food Safety LAVES - Institute of Apiculture | Lower Saxony State Office for Consumer Protection and Food Safety LAVES - Institute of Apiculture | 178 |
|  | beads (counting & speed beads) | all | - | - | 200 |
|  | no pollen | anther | anther samples | - | 200 |
|  | no pollen | honey | honey samples | - | 200 |
| prediction | *Brassica* | honey | University of Hohenheim - State Institute of Bee Research | University of Hohenheim - State Institute of Bee Research | - |
|  | *Brassica* | honey | University of Hohenheim - State Institute of Bee Research | University of Hohenheim - State Institute of Bee Research | - |
|  | *Brassica* | honey | University of Hohenheim - State Institute of Bee Research | University of Hohenheim - State Institute of Bee Research | - |
|  | *Castanea* | honey | University of Hohenheim - State Institute of Bee Research | University of Hohenheim - State Institute of Bee Research | - |
|  | *Castanea* | honey | University of Hohenheim - State Institute of Bee Research | University of Hohenheim - State Institute of Bee Research | - |
|  | *Castanea* | honey | University of Hohenheim - State Institute of Bee Research | University of Hohenheim - State Institute of Bee Research | - |
|  | *Centaurea* | honey | Lower Saxony State Office for Consumer Protection and Food Safety LAVES - Institute of Apiculture | Lower Saxony State Office for Consumer Protection and Food Safety LAVES - Institute of Apiculture | - |
|  | *Helianthus* | honey | University of Hohenheim - State Institute of Bee Research | University of Hohenheim - State Institute of Bee Research | - |
|  | *Phacelia* | honey | University of Hohenheim - State Institute of Bee Research | University of Hohenheim - State Institute of Bee Research | - |
|  | *Tilia* | honey | Lower Saxony State Office for Consumer Protection and Food Safety LAVES - Institute of Apiculture | Lower Saxony State Office for Consumer Protection and Food Safety LAVES - Institute of Apiculture | - |
|  | *Tilia* | honey | Lower Saxony State Office for Consumer Protection and Food Safety LAVES - Institute of Apiculture | Lower Saxony State Office for Consumer Protection and Food Safety LAVES - Institute of Apiculture | - |
|  | *Tilia* | honey | Lower Saxony State Office for Consumer Protection and Food Safety LAVES - Institute of Apiculture | Lower Saxony State Office for Consumer Protection and Food Safety LAVES - Institute of Apiculture | - |
|  | *Tilia* | honey | Lower Saxony State Office for Consumer Protection and Food Safety LAVES - Institute of Apiculture | Lower Saxony State Office for Consumer Protection and Food Safety LAVES - Institute of Apiculture | - |

**Supplementary Table S2**: Overview of all polyfloral honey samples, including origin and microscopic pollen analysis.

| **polyfloral honey** | **provided from** | **microscopic pollen analysis carried out by** |
| --- | --- | --- |
| summer blossom honey | Hans-Martin Schlegel | Lower Saxony State Office for Consumer Protection and Food Safety LAVES - Institute of Apiculture |
| summer blossom honey | German Beekeepers' Association (DIB e.V.) | Lower Saxony State Office for Consumer Protection and Food Safety LAVES - Institute of Apiculture |
| summer blossom honey | German Beekeepers' Association (DIB e.V.) | Lower Saxony State Office for Consumer Protection and Food Safety LAVES - Institute of Apiculture |
| summer blossom honey | German Beekeepers' Association (DIB e.V.) | Lower Saxony State Office for Consumer Protection and Food Safety LAVES - Institute of Apiculture |
| fir honey with *Castanea sativa* and *Brassica napus* | Lower Saxony State Office for Consumer Protection and Food Safety LAVES - Institute of Apiculture | Lower Saxony State Office for Consumer Protection and Food Safety LAVES - Institute of Apiculture |
| spring blossom honey | beekeeper from Leipzig | Lower Saxony State Office for Consumer Protection and Food Safety LAVES - Institute of Apiculture |
| spring blossom honey with *Brassica napus* | Mr. Börngen | Lower Saxony State Office for Consumer Protection and Food Safety LAVES - Institute of Apiculture |
| summer blossom honey | Mr. Börngen | Lower Saxony State Office for Consumer Protection and Food Safety LAVES - Institute of Apiculture |
| spring blossom honey | Mr. Börngen | Lower Saxony State Office for Consumer Protection and Food Safety LAVES - Institute of Apiculture |
| greek pure flower honey | beekeeper from Greece | Lower Saxony State Office for Consumer Protection and Food Safety LAVES - Institute of Apiculture |

**Supplementary Table S3**: Overview of category and description (according to IDEAS 6.2 User's Manual), of the twenty pollen features.

| **feature** | **category** | **description** |
| --- | --- | --- |
| Area | size | The number of microns squared in a mask is equal to the Area. The number of pixels is converted to µm^2^ (1 pixel = 0.25 µm^2^). |
| Brightfield Intensity (Ch 1) | signal strength | Sum of background subtracted pixel values within masked area of image for channel 01. |
| Circularity | shape | Measures degree of mask’s deviation from a circle by calculating the average distance of the object boundary from its centre divided by the variation of this distance. |
| Compactness | shape | Measures degree of how well object is packed together (intensity weighted for all pixels in a mask). |
| Diameter | size | Diameter of the circle that has the same area as the object. Highly dependent on a close fitting mask and roundness of cell. Formula: 2* $\sqrt{\frac{Area}{\pi}}$ |
| Elongatedness | shape | Ratio of height over width of the object's bounding box. |
| Green Fluorescence Intensity (Ch 2) | signal strength | Sum of background subtracted pixel values within masked area of image for channel 02. |
| Height | size | Number of microns of the longer side, when using the bounding rectangle. |
| Length | size | Measures longest part of object based on input mask. |
| Major Axis | size | Longest dimension of an ellipse of best fit. |
| Minor Axis | size | Narrowest dimension of the ellipse of best fit. |
| Orange Fluorescence Intensity (Ch 4) | signal strength | Sum of background subtracted pixel values within masked area of image for channel 04. |
| Perimeter | size | Measures the boundary length of the mask in the number of microns. |
| Red Fluorescence Intensity (Ch 5) | signal strength | Sum of background subtracted pixel values within masked area of image for channel 05. |
| Scatter Intensity (Ch 6) | signal strength | Sum of background subtracted pixel values within masked area of image for channel 06. |
| Symmetry 2 | shape | Measures tendency of the object to have a single axis of elongation and therefore 2 lobes. |
| Symmetry 3 | shape | Measures tendency of the object to have a three-fold axis of symmetry and therefore 3 lobes. |
| Symmetry 4 | shape | Measures tendency of the object to have a four-fold axis of symmetry and therefore 4 lobes. |
| Width | size | Number of microns of the smaller side, when using a bounding rectangle. |
| Yellow Fluorescence Intensity (Ch 3) | signal strength | Sum of background subtracted pixel values within masked area of image for channel 03. |

**Supplementary Table S4**: Overview of the hyperparameter ranges for the parameter optimisation.

| hyperparameter | range/options |
| --- | --- |
| learning rate | log-uniform distribution between 1e-5 and 1e-2 |
| optimizer | choice between SGD and AdamW |
| batch size | options of 32, 64, 128, or 256 |
| random erase probability | uniform distribution between 0.01 and 0.9 |
| mixup alpha | uniform distribution between 0 and 0.9 |
| cutMix alpha | uniform distribution between 0 and 0.9 |
| synaptic Scaling | uniform distribution between 0 and 0.1 |
| resize | uniform distribution 32 x 32 to 512 x 512 |
| random crop | uniform distribution 32x32 to 512 x 512 |

**Supplementary Table S5**: Identified pollen count in monofloral honeys (*Brassica, Castanea, Centaurea, Helianthus, Phacelia, Tilia*) for the bCNN approach obtained with all configurations.

| **configuration** | **true_class** | ***Brassica*** | ***Castanea*** | ***Centaurea*** | ***Helianthus*** | ***Phacelia*** | ***Tilia*** | **beads** | **no.pollen** | **n_files** |
| --- | --- | --- | --- | --- | --- | --- | --- | --- | --- | --- |
| OoD_BF | *Brassica* | 952 | 0 | 66 | 263 | 1 | 94 | 0 | 0 | 3 |
| OoD_BF | *Castanea* | 2 | 1832 | 0 | 1 | 323 | 0 | 0 | 12 | 3 |
| OoD_BF | *Centaurea* | 1 | 0 | 79 | 1 | 0 | 76 | 0 | 0 | 1 |
| OoD_BF | *Helianthus* | 0 | 0 | 5 | 480 | 0 | 0 | 0 | 0 | 1 |
| OoD_BF | *Phacelia* | 0 | 1 | 0 | 8 | 103 | 0 | 0 | 10 | 1 |
| OoD_BF | *Tilia* | 0 | 0 | 12 | 0 | 2 | 59 | 0 | 0 | 4 |
| OoD_all | *Brassica* | 0 | 0 | 1304 | 0 | 65 | 7 | 0 | 0 | 3 |
| OoD_all | *Castanea* | 0 | 13 | 1 | 0 | 2135 | 19 | 0 | 2 | 3 |
| OoD_all | *Centaurea* | 0 | 0 | 156 | 0 | 0 | 1 | 0 | 0 | 1 |
| OoD_all | *Helianthus* | 0 | 0 | 476 | 3 | 0 | 6 | 0 | 0 | 1 |
| OoD_all | *Phacelia* | 0 | 0 | 104 | 0 | 18 | 0 | 0 | 0 | 1 |
| OoD_all | *Tilia* | 0 | 0 | 53 | 2 | 1 | 17 | 0 | 0 | 4 |
| ID_BF | *Brassica* | 1333 | 1 | 0 | 29 | 1 | 10 | 0 | 2 | 3 |
| ID_BF | *Castanea* | 0 | 2161 | 0 | 0 | 0 | 0 | 0 | 9 | 3 |
| ID_BF | *Centaurea* | 10 | 0 | 133 | 1 | 0 | 13 | 0 | 0 | 1 |
| ID_BF | *Helianthus* | 2 | 0 | 0 | 464 | 0 | 0 | 0 | 19 | 1 |
| ID_BF | *Phacelia* | 0 | 5 | 0 | 0 | 117 | 0 | 0 | 0 | 1 |
| ID_BF | *Tilia* | 1 | 0 | 5 | 0 | 0 | 67 | 0 | 0 | 4 |
| ID_all | *Brassica* | 1369 | 1 | 2 | 1 | 0 | 3 | 0 | 0 | 3 |
| ID_all | *Castanea* | 0 | 2162 | 0 | 0 | 3 | 1 | 0 | 4 | 3 |
| ID_all | *Centaurea* | 3 | 0 | 135 | 5 | 0 | 14 | 0 | 0 | 1 |
| ID_all | *Helianthus* | 24 | 0 | 4 | 449 | 0 | 8 | 0 | 0 | 1 |
| ID_all | *Phacelia* | 0 | 13 | 0 | 0 | 99 | 0 | 0 | 1 | 1 |
| ID_all | *Tilia* | 1 | 0 | 3 | 3 | 0 | 66 | 0 | 0 | 4 |
| MD_BF | *Brassica* | 1300 | 1 | 3 | 34 | 0 | 10 | 0 | 28 | 3 |
| MD_BF | *Castanea* | 0 | 2147 | 0 | 0 | 6 | 0 | 0 | 17 | 3 |
| MD_BF | *Centaurea* | 12 | 1 | 129 | 1 | 0 | 14 | 0 | 0 | 1 |
| MD_BF | *Helianthus* | 43 | 0 | 7 | 422 | 0 | 9 | 0 | 4 | 1 |
| MD_BF | *Phacelia* | 0 | 1 | 0 | 0 | 109 | 0 | 0 | 12 | 1 |
| MD_BF | *Tilia* | 4 | 1 | 5 | 0 | 0 | 63 | 0 | 0 | 4 |
| MD_all | *Brassica* | 1351 | 2 | 16 | 6 | 0 | 1 | 0 | 0 | 3 |
| MD_all | *Castanea* | 0 | 2168 | 0 | 0 | 2 | 0 | 0 | 0 | 3 |
| MD_all | *Centaurea* | 3 | 0 | 145 | 6 | 0 | 3 | 0 | 0 | 1 |
| MD_all | *Helianthus* | 8 | 0 | 1 | 469 | 0 | 7 | 0 | 0 | 1 |
| MD_all | *Phacelia* | 0 | 0 | 3 | 1 | 115 | 0 | 0 | 3 | 1 |
| MD_all | *Tilia* | 1 | 0 | 8 | 3 | 0 | 61 | 0 | 0 | 4 |

**Supplementary Table S6**: Identified pollen count in monofloral honeys (*Brassica, Castanea, Centaurea, Helianthus, Phacelia, Tilia*) for the cCNN approach obtained with all configurations.

| **configuration** | **true_class_name** | ***Brassica*** | ***Castanea*** | ***Centaurea*** | ***Helianthus*** | ***Phacelia*** | ***Tilia*** | **beads** | **no.pollen** | **n_files** |
| --- | --- | --- | --- | --- | --- | --- | --- | --- | --- | --- |
| OoD_BF | *Brassica* | 759 | 1 | 104 | 271 | 1 | 235 | 0 | 13 | 3 |
| OoD_BF | *Castanea* | 10 | 1778 | 1 | 4 | 226 | 9 | 0 | 142 | 3 |
| OoD_BF | *Centaurea* | 12 | 0 | 79 | 1 | 1 | 65 | 0 | 0 | 1 |
| OoD_BF | *Helianthus* | 1 | 1 | 0 | 483 | 1 | 0 | 0 | 1 | 1 |
| OoD_BF | *Phacelia* | 0 | 8 | 0 | 0 | 102 | 1 | 0 | 2 | 1 |
| OoD_BF | *Tilia* | 10 | 0 | 8 | 0 | 1 | 54 | 0 | 0 | 4 |
| OoD_all | *Brassica* | 90 | 298 | 762 | 92 | 16 | 119 | 4 | 3 | 3 |
| OoD_all | *Castanea* | 0 | 269 | 1608 | 2 | 166 | 18 | 0 | 107 | 3 |
| OoD_all | *Centaurea* | 0 | 0 | 114 | 39 | 0 | 5 | 0 | 0 | 1 |
| OoD_all | *Helianthus* | 0 | 3 | 92 | 253 | 2 | 137 | 0 | 0 | 1 |
| OoD_all | *Phacelia* | 0 | 41 | 4 | 0 | 66 | 0 | 0 | 2 | 1 |
| OoD_all | *Tilia* | 1 | 0 | 31 | 5 | 0 | 36 | 0 | 0 | 4 |
| ID_BF | *Brassica* | 1354 | 2 | 0 | 23 | 0 | 3 | 0 | 2 | 3 |
| ID_BF | *Castanea* | 7 | 2155 | 1 | 0 | 6 | 1 | 0 | 0 | 3 |
| ID_BF | *Centaurea* | 22 | 0 | 118 | 0 | 0 | 17 | 0 | 1 | 1 |
| ID_BF | *Helianthus* | 0 | 3 | 0 | 484 | 0 | 0 | 0 | 0 | 1 |
| ID_BF | *Phacelia* | 1 | 8 | 0 | 0 | 101 | 2 | 0 | 1 | 1 |
| ID_BF | *Tilia* | 7 | 0 | 6 | 0 | 0 | 60 | 0 | 0 | 4 |
| ID_all | *Brassica* | 1374 | 0 | 3 | 1 | 2 | 1 | 0 | 3 | 3 |
| ID_all | *Castanea* | 2 | 2122 | 0 | 0 | 30 | 3 | 0 | 13 | 3 |
| ID_all | *Centaurea* | 3 | 0 | 153 | 1 | 0 | 1 | 0 | 0 | 1 |
| ID_all | *Helianthus* | 10 | 0 | 1 | 455 | 0 | 17 | 0 | 4 | 1 |
| ID_all | *Phacelia* | 0 | 5 | 0 | 0 | 105 | 0 | 0 | 3 | 1 |
| ID_all | *Tilia* | 1 | 1 | 1 | 1 | 0 | 69 | 0 | 0 | 4 |
| MD_BF | *Brassica* | 1361 | 0 | 4 | 15 | 0 | 3 | 0 | 1 | 3 |
| MD_BF | *Castanea* | 5 | 2161 | 0 | 0 | 2 | 1 | 0 | 1 | 3 |
| MD_BF | *Centaurea* | 19 | 0 | 123 | 0 | 0 | 16 | 0 | 0 | 1 |
| MD_BF | *Helianthus* | 0 | 1 | 0 | 486 | 0 | 0 | 0 | 0 | 1 |
| MD_BF | *Phacelia* | 0 | 10 | 0 | 0 | 102 | 0 | 0 | 1 | 1 |
| MD_BF | *Tilia* | 4 | 1 | 4 | 0 | 0 | 64 | 0 | 0 | 4 |
| MD_all | *Brassica* | 1373 | 0 | 5 | 3 | 3 | 0 | 0 | 0 | 3 |
| MD_all | *Castanea* | 1 | 2145 | 0 | 1 | 18 | 1 | 0 | 4 | 3 |
| MD_all | *Centaurea* | 3 | 0 | 153 | 1 | 0 | 1 | 0 | 0 | 1 |
| MD_all | *Helianthus* | 4 | 0 | 3 | 467 | 0 | 11 | 0 | 2 | 1 |
| MD_all | *Phacelia* | 1 | 6 | 0 | 0 | 106 | 0 | 0 | 0 | 1 |
| MD_all | Tilia | 0 | 0 | 1 | 1 | 0 | 71 | 0 | 0 | 4 |

**Supplementary Table S7**: Identified pollen count in monofloral honeys (*Brassica, Castanea, Centaurea, Helianthus, Phacelia, Tilia*) for the RF approach obtained with all configurations.

| **configuration** | **true_class_name** | ***Brassica*** | ***Castanea*** | ***Centaurea*** | ***Helianthus*** | ***Phacelia*** | ***Tilia*** | **beads** | **no pollen** | **n_files** |
| --- | --- | --- | --- | --- | --- | --- | --- | --- | --- | --- |
| OoD_BF | *Brassica* | 652 | 0 | 227 | 20 | 7 | 462 | 0 | 0 | 3 |
| OoD_BF | *Castanea* | 0 | 1559 | 0 | 0 | 166 | 0 | 0 | 0 | 3 |
| OoD_BF | *Centaurea* | 1 | 0 | 45 | 25 | 0 | 81 | 0 | 0 | 1 |
| OoD_BF | *Helianthus* | 0 | 0 | 7 | 270 | 0 | 188 | 0 | 0 | 1 |
| OoD_BF | *Phacelia* | 63 | 2 | 0 | 0 | 41 | 0 | 0 | 0 | 1 |
| OoD_BF | *Tilia* | 2 | 0 | 5 | 13 | 0 | 53 | 0 | 0 | 4 |
| OoD_all | *Brassica* | 347 | 0 | 741 | 0 | 13 | 283 | 0 | 0 | 3 |
| OoD_all | *Castanea* | 0 | 195 | 0 | 0 | 1945 | 0 | 0 | 0 | 3 |
| OoD_all | *Centaurea* | 0 | 0 | 103 | 0 | 0 | 55 | 0 | 0 | 1 |
| OoD_all | *Helianthus* | 0 | 0 | 347 | 1 | 0 | 139 | 0 | 0 | 1 |
| OoD_all | *Phacelia* | 50 | 0 | 0 | 0 | 59 | 4 | 0 | 0 | 1 |
| OoD_all | *Tilia* | 0 | 0 | 38 | 7 | 0 | 28 | 0 | 0 | 4 |
| ID_BF | *Brassica* | 1243 | 19 | 21 | 4 | 0 | 69 | 0 | 0 | 3 |
| ID_BF | *Castanea* | 0 | 2113 | 0 | 0 | 1 | 0 | 0 | 0 | 3 |
| ID_BF | *Centaurea* | 10 | 3 | 102 | 23 | 0 | 20 | 0 | 0 | 1 |
| ID_BF | *Helianthus* | 5 | 10 | 50 | 404 | 0 | 10 | 0 | 0 | 1 |
| ID_BF | *Phacelia* | 0 | 11 | 0 | 0 | 97 | 0 | 0 | 0 | 1 |
| ID_BF | *Tilia* | 5 | 0 | 7 | 5 | 0 | 56 | 0 | 0 | 4 |
| ID_all | *Brassica* | 1331 | 31 | 0 | 3 | 0 | 18 | 0 | 0 | 3 |
| ID_all | *Castanea* | 0 | 2158 | 0 | 0 | 0 | 0 | 0 | 0 | 3 |
| ID_all | *Centaurea* | 1 | 0 | 138 | 11 | 0 | 8 | 0 | 0 | 1 |
| ID_all | *Helianthus* | 0 | 8 | 22 | 431 | 0 | 26 | 0 | 0 | 1 |
| ID_all | *Phacelia* | 1 | 15 | 0 | 0 | 97 | 0 | 0 | 0 | 1 |
| ID_all | *Tilia* | 5 | 0 | 1 | 7 | 0 | 60 | 0 | 0 | 4 |
| MD_BF | *Brassica* | 1194 | 26 | 26 | 2 | 0 | 115 | 0 | 0 | 3 |
| MD_BF | *Castanea* | 0 | 2130 | 0 | 0 | 5 | 0 | 0 | 0 | 3 |
| MD_BF | *Centaurea* | 3 | 2 | 95 | 26 | 0 | 31 | 0 | 0 | 1 |
| MD_BF | *Helianthus* | 2 | 10 | 52 | 413 | 0 | 4 | 0 | 0 | 1 |
| MD_BF | *Phacelia* | 0 | 9 | 0 | 0 | 100 | 0 | 0 | 0 | 1 |
| MD_BF | *Tilia* | 3 | 0 | 3 | 8 | 0 | 59 | 0 | 0 | 4 |
| MD_all | *Brassica* | 1299 | 41 | 5 | 2 | 1 | 33 | 0 | 0 | 3 |
| MD_all | *Castanea* | 0 | 2169 | 0 | 0 | 1 | 0 | 0 | 0 | 3 |
| MD_all | *Centaurea* | 1 | 0 | 141 | 5 | 0 | 11 | 0 | 0 | 1 |
| MD_all | *Helianthus* | 0 | 8 | 22 | 434 | 0 | 23 | 0 | 0 | 1 |
| MD_all | *Phacelia* | 0 | 9 | 0 | 0 | 104 | 0 | 0 | 0 | 1 |
| MD_all | *Tilia* | 2 | 0 | 3 | 9 | 0 | 59 | 0 | 0 | 4 |

| **approach** | **true_class** | ***Brassica*** | ***Castanea*** | ***Centaurea*** | ***Helianthus*** | ***Phacelia*** | ***Tilia*** | **no.pollen** | **beads** | | **Rosaceae** | **n_files** |
| --- | --- | --- | --- | --- | --- | --- | --- | --- | --- | --- | --- | --- |
| bCNN | *Brassica* | 4016 | 124 | 44 | 63 | 2 | 186 | 27751 | 0 | 70 | | 3 |
|  | *Castanea* | 166 | 6860 | 35 | 27 | 39 | 134 | 25005 | 1 | 57 | | 3 |
|  | *Centaurea* | 321 | 406 | 410 | 9 | 117 | 259 | 1277 | 0 | 768 | | 1 |
|  | *Helianthus* | 54 | 14 | 1 | 482 | 0 | 13 | 401 | 0 | 9 | | 1 |
|  | *Phacelia* | 16 | 51 | 7 | 1 | 110 | 5 | 428 | 0 | 14 | | 1 |
|  | *Tilia* | 184 | 699 | 31 | 58 | 409 | 314 | 6146 | 1 | 119 | | 4 |
| cCNN | *Brassica* | 4306 | 4022 | 19 | 33 | 14 | 169 | 245 | 23850 | 91 | | 3 |
|  | *Castanea* | 600 | 13980 | 14 | 37 | 131 | 187 | 22 | 17491 | 136 | | 3 |
|  | *Centaurea* | 403 | 571 | 521 | 173 | 116 | 216 | 10 | 826 | 854 | | 1 |
|  | *Helianthus* | 47 | 98 | 1 | 530 | 4 | 27 | 2 | 298 | 30 | | 1 |
|  | *Phacelia* | 24 | 115 | 8 | 1 | 146 | 7 | 2 | 349 | 10 | | 1 |
|  | *Tilia* | 196 | 1879 | 26 | 36 | 268 | 459 | 33 | 5154 | 167 | | 4 |
| RF | *Brassica* | 3095 | 757 | 63 | 365 | 222 | 309 | 0 | 0 | 0 | | 3 |
|  | *Castanea* | 139 | 7442 | 7 | 113 | 354 | 145 | 0 | 0 | 0 | | 3 |
|  | *Centaurea* | 253 | 525 | 325 | 122 | 362 | 194 | 0 | 0 | 0 | | 1 |
|  | *Helianthus* | 9 | 59 | 35 | 490 | 16 | 39 | 0 | 0 | 0 | | 1 |
|  | *Phacelia* | 10 | 53 | 3 | 13 | 131 | 6 | 0 | 0 | 0 | | 1 |
|  | *Tilia* | 196 | 674 | 22 | 75 | 699 | 223 | 0 | 0 | 0 | | 4 |

**Supplementary Table S8**: Identified pollen count in monofloral honeys (*Brassica, Castanea, Centaurea, Helianthus, Phacelia, Tilia*) for the three machine learning approaches obtained with the final configuration.

**Supplementary Table S9**: Overview of predicted pollen counts for the entire measurement, based on the final configuration of the bCNN, along with the corresponding measurement time for all honey samples.

| **honey sample** | **number of predicted pollen in total measurement** | **measurement time in min:sec** |
| --- | --- | --- |
| *Brassica napus* (ID: 43) | 1,690 | 09:59 |
| *Brassica napus* (ID: 58) | 1,181 | 01:02 |
| *Brassica napus* (ID: 61) | 1,564 | 09:59 |
| *Castanea sativa* (ID: 62) | 2,142 | 09:59 |
| *Castanea sativa* (ID: 73) | 1,698 | 08:05 |
| *Castanea sativa* (ID: 75) | 3,421 | 01:11 |
| *Centaurea cyanus* (ID: 81) | 1,522 | 09:59 |
| *Helianthus annuus* (ID: 71) | 564 | 08:36 |
| *Phacelia tanacetifolia* (ID: 59) | 190 | 19:58 |
| *Tilia* sp. (ID: 83) | 430 | 19:58 |
| *Tilia* sp. (ID: 85) | 777 | 19:58 |
| *Tilia* sp. (ID: 86) | 330 | 19:58 |
| *Tilia* sp. (ID: 87) | 158 | 19:58 |
| summer blossom honey (ID: 3) | 129 | 09:59 |
| summer blossom honey (ID: 4) | 1,510 | 09:59 |
| summer blossom honey  (ID: 18) | 5,044 | 02:28 |
| summer blossom honey  (ID: 20) | 619 | 09:59 |
| fir honey with *Castanea sativa* and *Brassica napus* (ID: 33) | 2,008 | 09:59 |
| spring blossom honey (ID: 34) | 7,734 | 09:59 |
| spring blossom honey with *Brassica napus* (ID: 37) | 1,834 | 09:59 |
| summer blossom honey  (ID: 38) | 100 | 09:59 |
| spring blossom honey (ID: 40) | 1,823 | 09:59 |
| greek pure flower honey  (ID: 77) | 3,713 | 09:59 |

| **configuration** |  | out-of-domain brightfield | | | out-of-domain  with all channels | | | in-domain   brightfield | | | in-domain  with all channels | | | mixed-domain  brightfield | | | | mixed-domain  with all channels | | | |
| --- | --- | --- | --- | --- | --- | --- | --- | --- | --- | --- | --- | --- | --- | --- | --- | --- | --- | --- | --- | --- | --- |
| **pollen matrix** |  | anthers | | | anthers | | | honey | | | honey | | | anthers & honey | | | | anthers & honey | | | |
| **channels** |  | 2  (brightfield) | | | 12  (2 brightfield, 1 sidescatter,  10 fluorescent) | | | 2  (brightfield) | | | 12  (2 brightfield, 1 sidescatter,  10 fluorescent) | | | 2  (brightfield) | | | | 12  (2 brightfield, 1 sidescatter,  10 fluorescent) | | | |
| **identification accuracy per pollen classes for each individual honey samples**  **in %** | target pollen | bCNN | cCNN | RF | bCNN | cCNN | RF | bCNN | cCNN | RF | bCNN | cCNN | RF | bCNN | cCNN | RF | bCNN | | cCNN | RF |  |
|  | *Brassica* | 91.6 | 74.7 | 69.7 | 0 | 7.3 | 32.1 | 98 | 100 | 94.3 | 99.7 | 99.7 | 99.2 | 99 | 99.7 | 90.3 | 98.9 | | 99.4 | 98 |  |
|  | *Brassica* | 67 | 56.2 | 51.8 | 0 | 6.3 | 28.5 | 97.3 | 98.6 | 90.2 | 99.7 | 99.6 | 94.3 | 96.4 | 98.9 | 84.7 | 98.4 | | 99 | 91.6 |  |
|  | *Brassica* | 55.7 | 38.5 | 25.3 | 0 | 5.1 | 11.3 | 94.9 | 95.3 | 88.1 | 99.3 | 99 | 92.4 | 94.4 | 96.6 | 83.9 | 96.3 | | 99 | 89.9 |  |
|  | *Castanea* | 85.9 | 88.8 | 89.3 | 1.3 | 17.7 | 3.2 | 100 | 99.5 | 100 | 99.9 | 99.7 | 100 | 100 | 100 | 99.9 | 100 | | 99.3 | 100 |  |
|  | *Castanea* | 84.5 | 87.9 | 89.2 | 0.6 | 13.5 | 2.3 | 100 | 99.2 | 100 | 99.8 | 98 | 100 | 99.7 | 99.7 | 99.9 | 99.9 | | 99.2 | 100 |  |
|  | *Castanea* | 84.3 | 85.6 | 88.4 | 0.1 | 10.2 | 1.0 | 100 | 99.2 | 100 | 99.8 | 97.6 | 100 | 99.4 | 99.3 | 99.4 | 99.8 | | 98.7 | 99.8 |  |
|  | *Centaurea* | 50.3 | 50 | 27.6 | 99.4 | 72.2 | 64.6 | 84.7 | 75.2 | 61.8 | 86 | 96.8 | 88.6 | 82.2 | 77.8 | 58 | 92.4 | | 96.8 | 89.9 |  |
|  | *Helianthus* | 99 | 99.4 | 61 | 0.6 | 52 | 0.2 | 99.6 | 99.4 | 84.2 | 92.6 | 94.2 | 87.3 | 87.7 | 99.8 | 86.5 | 96.7 | | 96.3 | 88.7 |  |
|  | *Phacelia* | 92 | 91.9 | 38.7 | 14.8 | 59.5 | 52.2 | 95.9 | 90.2 | 90.7 | 88.4 | 95.5 | 85.8 | 99.1 | 91.1 | 91.7 | 96.6 | | 93.8 | 92.9 |  |
|  | *Tilia* | 85.7 | 80 | 85.7 | 33.3 | 73.3 | 42.9 | 100 | 100 | 100 | 100 | 100 | 90 | 100 | 95.2 | 100 | 100 | | 100 | 85.7 |  |
|  | *Tilia* | 83.3 | 76.7 | 76.7 | 23.3 | 53.3 | 33.3 | 95.2 | 100 | 80 | 96.7 | 100 | 85.7 | 90.5 | 93.3 | 83.3 | 96.7 | | 100 | 80 |  |
|  | *Tilia* | 80 | 71.4 | 66.7 | 14.3 | 38.1 | 33.3 | 93.3 | 73.3 | 76.2 | 85.7 | 93.3 | 80 | 86.7 | 85.7 | 80 | 80 | | 100 | 80 |  |
|  | *Tilia* | 76.2 | 66.7 | 66.7 | 13.3 | 14.3 | 26.7 | 86.7 | 66.7 | 73.3 | 80 | 90 | 71.4 | 80 | 80 | 76.2 | 61.9 | | 93.3 | 76.2 |  |
| **total (mean)** | | **79.7** | **74.4** | **64.4** | **15.5** | **32.5** | **25.5** | **95.8** | **92.0** | **87.6** | **94.4** | **97.2** | **90.4** | **93.5** | **93.6** | **87.2** | **93.7** | | **98.1** | **90.2** |  |

**Supplementary Table S10**: Overview of the identification results obtained with the bCNN, cCNN and RF for each individual honey sample. The total identification accuracy is marked in bold.

| **polyfloral honey** | ***Brassica*** | ***Castanea*** | ***Phacelia*** | **Rosaceae** | ***Tilia*** | **beads** | **no.pollen** | **Total_Object_Count** | **Total_Pollen** |
| --- | --- | --- | --- | --- | --- | --- | --- | --- | --- |
| summer blossom honey | 38 | 43 | 13 | 15 | 16 | 875 | 477 | 1481 | 129 |
| summer blossom honey | 245 | 881 | 60 | 196 | 98 | 1548 | 6755 | 9813 | 1510 |
| summer blossom honey | 1387 | 1963 | 291 | 925 | 342 | 186 | 37414 | 42644 | 5044 |
| summer blossom honey | 52 | 468 | 32 | 32 | 22 | 1920 | 2262 | 4801 | 619 |
| fir honey with *Castanea sativa* & *Brassica napus* | 162 | 1515 | 9 | 109 | 119 | 9 | 7159 | 9176 | 2008 |
| spring blossom honey | 3961 | 336 | 246 | 1331 | 660 | 1 | 1563 | 9298 | 7734 |
| spring blossom honey with *Brassica napus* | 907 | 50 | 0 | 43 | 615 | 0 | 163 | 1997 | 1834 |
| summer blossom honey | 17 | 45 | 11 | 10 | 12 | 0 | 236 | 336 | 100 |
| spring blossom honey | 1017 | 324 | 15 | 134 | 270 | 0 | 803 | 2626 | 1823 |
| greek pure flower honey | 636 | 1950 | 39 | 490 | 373 | 4 | 11164 | 14881 | 3713 |

**Supplementary Table S11:** Identified pollen count in polyfloral honeys for the bCNN approach obtained with the final configuration (mixed-domain configuration with all channels).

**Supplementary Table S12**: Overview of the coefficients of variation (CVs) for pollen from anthers and honey for the features “Red Fluorescence Intensity” and “Size”. CVs were calculated as the ratio of the standard deviation to the mean for each species and feature.

| **species** | **feature** | **cv anthers** | **cv honey** | **cv ratio anthers/honey** | **cv ratio honey/anthers** |
| --- | --- | --- | --- | --- | --- |
| *Brassica* | Red Fluorescence Intensity | 0.99 | 0.19 | 5.21 | 0.19 |
| *Brassica* | Size | 0.05 | 0.16 | 0.29 | 3.48 |
| *Castanea* | Red Fluorescence Intensity | 0.26 | 0.20 | 1.26 | 0.79 |
| *Castanea* | Size | 0.05 | 0.14 | 0.38 | 2.66 |
| *Centaurea* | Red Fluorescence Intensity | 0.23 | 0.21 | 1.07 | 0.93 |
| *Centaurea* | Size | 0.06 | 0.08 | 0.72 | 1.38 |
| *Helianthus* | Red Fluorescence Intensity | 0.12 | 0.25 | 0.49 | 2.05 |
| *Helianthus* | Size | 0.05 | 0.07 | 0.65 | 1.55 |
| *Phacelia* | Red Fluorescence Intensity | 0.23 | 0.46 | 0.51 | 1.97 |
| *Phacelia* | Size | 0.05 | 0.09 | 0.56 | 1.79 |
| *Tilia* | Red Fluorescence Intensity | 0.19 | 0.99 | 0.19 | 5.28 |
| *Tilia* | Size | 0.07 | 0.09 | 0.77 | 1.30 |

**Supplementary Table S13**: Post hoc results for multidimensional feature data resemblance matrix. The asterisks stand for the following significances: ‘***’ 0.001 ‘**’ 0.01 ‘*’ 0.05.

| pairs | feature | df | t.ratio | p-value |
| --- | --- | --- | --- | --- |
| anthers vs corbiculae | size | 6 | -0.300 | 0.9521 |
| anthers vs honey | size | 6 | -2.832 | 0.0670 |
| corbiculae vs honey | size | 6 | -2.532 | 0.0979 |
| anthers vs corbiculae | red fluorescence intensity | 6 | -1.109 | 0.5433 |
| anthers vs honey | red fluorescence intensity | 6 | -23.947 | P < 0.0001 *** |
| corbiculae vs honey | red fluorescence intensity | 6 | -22.838 | P < 0.0001 *** |

**Supplementary Figures**

**Supplemental Figure 1**: Bland-Altman plot of the identification results for the complete monofloral honeys based on the final configuration for all three machine learning approaches (left-bCNN, middle-cCNN, right-RF).

**Supplemental Figure 2**: Density and dot plot for two selected *Castanea sativa* pollen features from different pollen sources. The features shown are pollen size and red fluorescence (intensity Ch05). The pollen grains were taken from three different plants/anthers (green tones), one corbiculae (orange/brown tones) and three different honeys (purple tones).
